# Trioctylamine in the Synthesis of Tris(trimethylsilyl)arsine-Based InAs Quantum Dots Prevents the Formation of Si-Based Byproducts

**DOI:** 10.1021/jacs.5c11775

**Published:** 2025-10-21

**Authors:** Satyaprakash Panda, Lutfan Sinatra, Khursand E. Yorov, Galih R. Suwito, Alexander Bessonov, Marat Lutfullin, Luca Goldoni, Enrico Bergamaschi, Rosaria Brescia, Mirko Prato, Giorgio Divitini, Luca De Trizio, Liberato Manna

**Affiliations:** †Nanochemistry, ∥Materials Characterization, ⊥Molecular Modelling & Drug Discovery, #Electron Microscopy and ¶Electron Spectroscopy and Nanoscopy, □Chemistry Facility, 121451 Istituto Italiano di Tecnologia, Via Morego 30, 16163 Genova, Italy; ‡ Dipartimento di Chimica e Chimica Industriale, Università di Genova, 16146 Genova, Italy; § Quantum Solutions, Unit 8-Innovation Quarter, Oxford Technology Park, Kidlington OX5 1GN, Oxford, U.K.

## Abstract

To date, the best reported strategy to synthesize large colloidal indium arsenide (InAs) quantum dots (QDs) with precise control over size and size distribution consists of a seeded-growth synthesis that uses dioctylamine coupled with tris­(trimethylsilyl)­arsine as arsenic precursor and oleic acid as ligand. Here, we demonstrate through nuclear magnetic resonance studies that, in such an approach, dioctylamine and oleic acid condense at the high temperatures required for synthesis (>250 °C), releasing water as a byproduct. The released water, in turn, leads to the formation of trimethylsilanol, which subsequently condenses to form hexamethyldisiloxane and trimethylsilyl oleate. As a result, dioctylamine-based InAs QDs are contaminated, even after multiple washing steps, by both unbound trimethylsilyl oleate and bound trimethylsilyl-derived species. We further show that these issues can be solved by replacing dioctylamine with a tertiary amine, for example tri-n-octylamine, which prevents the formation of water and leads instead to clean InAs QDs. We also demonstrate that this modified procedure delivers InAs QDs with excellent control over their optical features, with excitonic absorption peaks as narrow as 50 meV (half-width at half-maximum) and peak-to-valley ratios (an important parameter for optoelectronic applications) as high as ∼2, representing a record value for InAs QDs.

## Graphical abstract

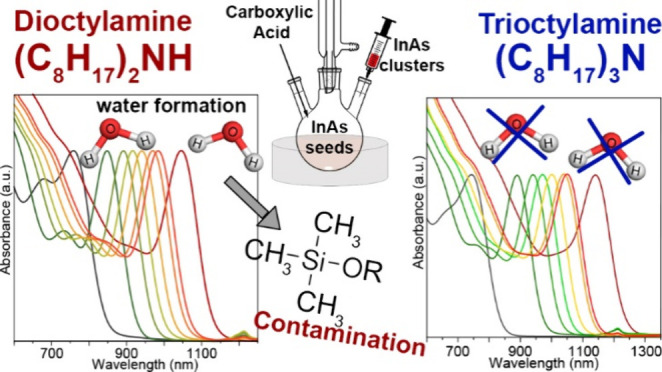

## Introduction

Colloidal quantum dots (QDs) that absorb and emit light in the infrared (IR) region of the spectrum are gaining increasing attention as active components for future IR consumer market optoelectronic technologies.^1,2^ The integration of IR QDs into well-established complementary metal-oxide semiconductor technologies offers a cost-efficient pathway for the commercialization of IR devices,^3,4^ including solar cells,^5,6^ light-emitting diodes,^7–10^ lasers,^11,12^ and sensors.^13^ These devices are essential for applications in areas such as security authentication,^14^ optogenetics,^15^ agricultural management,^16^ light fidelity communication,^17^ surveillance, automotive systems, night and cloud vision,^18^ object inspection, and optical communications.^19^

From a materials perspective, II–VI (Cd- and Hg-based)^20,21^ and IV–VI (Pb-based)^22,23^ semiconductors have long dominated the field of IR QDs, owing to their well-established synthesis procedures. However, the inherent toxicity of such compounds, which limits their use in consumer electronics, has driven the search toward safer alternatives.^24,25^ Promising IR materials that comply with the Restriction of Hazardous Substances (RoHS) directive include Ag- and Cu-based I–III–VI semiconductors (such as CuInSe_2_, CuInS_2_, and AgInSe_2_),^26^ Ag chalcogenides, and In-based III–V semiconductors such as InSb^27^ and InAs QDs. Among these, InAs QDs stand out due to their tunable bandgap, which can be adjusted from the visible range down to approximately 0.7 eV (1700 nm) through quantum confinement.^28–33^

To date, the most established synthesis protocol involves the use of tris­(trimethylsilyl)-As ((Me_3_Si)_3_As) or the analogous tris­(trimethylgermyl)-As.^34–37^ Such As precursors enable the synthesis of InAs QDs with a narrow size distribution, resulting in high-quality excitonic features: a half width at half-maximum (HWHM) of the first excitonic peak as low as ∼50 meV (at 1300 nm) and peak-to-valley (P/V) ratios as high as 1.6.^31^ The P/V ratio is defined as the ratio of the intensity at the first excitonic absorption peak to that at the minimum (i.e., valley) between this peak and the next one at higher energies. These optical parameters (i.e., a narrow HWHM and a high P/V ratio) are indicative of QDs with high optical and electrical quality, stemming from a narrow particle size distribution, reduced density of surface defects, and enhanced carrier confinement,^38^ and are crucial for developing high-performance IR optoelectronic applications, such as photodetectors, as they translate into efficient carrier extraction and low dark current, ultimately leading to improved overall detectivity and responsivity.^39^

In contrast, the alternative As precursors explored so far, including tris­(dimethylamino)­arsine,^32,33,40^ arsenic halides,^41,42^ and triphenylarsine,^43,44^ have delivered InAs QDs with poorer excitonic features, that is, HWHMs of ∼100 meV at best (at ∼800 nm)^45,46^ and low P/V ratios (close to 1).

The synthesis of InAs QDs via (Me_3_Si)_3_As typically involves its hot-injection into a reaction mixture containing indium acetate, oleic acid as the surfactant, and 1-octadecene as the noncoordinating solvent.^36,37^ The main issue associated with the use of (Me_3_Si)_3_As is its high reactivity, which triggers a rapid nucleation burst as a consequence of the hot-injection that quickly depletes the available monomers and leaves in the solution an insufficient concentration of monomers available for the subsequent growth of InAs QDs in a way that they can maintain a narrow size distribution. To overcome this limitation and grow larger InAs QDs while preserving a narrow size distribution, seeded-growth approaches have been developed that involve the continuous injection of precursors.^28,29^ The main improvements in this direction were achieved by Tamang et al., who introduced the use of InAs clusters as a single-source precursor, both to prepare InAs QDs and to grow them larger. These InAs clusters, defined as ultrasmall, atomically precise nanocrystals (characterized by a distinct absorption spectrum and a well-defined crystal structure), also referred to as magic-sized nanoclusters,^47^ were prepared at room temperature by reacting (Me_3_Si)_3_As with indium oleate and 1-octadecene. This method led to InAs QDs with an excitonic absorption peak reaching 1100 nm with a HWHM as low as ∼70 meV.

This strategy was soon improved by Song et al., who introduced dioctylamine (DOA) in all the reaction steps, even in the preparation of the clusters,^30^ to lower the reactivity of (Me_3_Si)_3_As. While the exact mechanism by which this moderation in reactivity is elicited is unclear, the use of DOA does enable a fine-tuning of the size of the starting InAs QDs and their subsequent growth upon the continuous addition of DOA-based InAs clusters, leading to QDs with a HWHM at 1100 nm as low as 57 meV.^30,48,49^ Careful adjustment of the injection rate of the InAs clusters was key to optimizing the growth of InAs QDs, so that the excitonic absorption peak could be shifted up to 1600 nm with an HWHM of ∼60 meV and a P/V ratio of 1.12. This strategy, despite being the best one reported so far, suffers from a drawback related to the copresence of a secondary amine (i.e., DOA) and a carboxylic acid (oleic acid), which are allowed to react at high temperatures (i.e., 300 °C) for hours. This can lead to their condensation reaction that produces an amide and water.^48^ The presence of water is detrimental for the growth of semiconductor QDs, as it can potentially lead to their surface oxidation and negatively affect their optical properties.^48,50^ This aspect has not yet been investigated, and the effects of in situ water release during the synthesis of InAs QDs are unknown. In this regard, our initial hypothesis was that byproducts such as In_2_O_3_ and/or the surface As_2_O_3_ observed in DOA-based approaches might be caused by the water that is formed in situ.^30,51^

In this work, to shed light onto these points, we have compared the optical and structural/chemical properties of InAs QDs synthesized using two analogous seeded growth approaches based on two different amines: DOA, which is the standard secondary amine used in the reports discussed earlier, and tri-n-octylamine (TOA), a tertiary amine, which represents the novel aspect of this study. We have chosen a tertiary amine since it is not able to condense with carboxylic acids, therefore it prevents any formation of water during the synthesis of the InAs QDs. Under the reaction conditions we devised, we could prepare InAs QDs with either DOA or TOA and achieve a fine control over QD size and size distribution, with the excitonic absorption peak tunable up to 1100 nm and featuring a HWHM as low as ∼50 meV and a P/V ratio around 2, the latter being a record value for these QDs. Despite their similar optimal optical properties, these two QD samples differed from each other in several aspects:

i) the purification of DOA-InAs QDs from the crude reaction mixture proved challenging and often led to the formation of a gel-like product and consequently to the loss of the entire sample. In contrast, TOA-InAs QDs could be easily purified using standard washing procedures;
ii) as evidenced by thermogravimetric analysis (TGA), DOA-based InAs QDs were characterized by a much higher weight loss than TOA-based ones, due to the presence of byproducts that could not be removed by washing;
iii) DOA-InAs, and not TOA-InAs QDs, were indeed contamintated by Si-based byproducts, as indicated by X-ray photoelectron spectroscopy (XPS) and corroborated by scanning transmission electron microscopy (STEM) energy dispersive spectroscopy (EDS).

To investigate the nature of these Si-based species, we carried out nuclear magnetic resonance (NMR) spectroscopy on DOA-based QDs, which indicated the presence of Me_3_Si-based compounds. Control experiments, in which (Me_3_Si)_3_As was allowed to react at 250 °C with DOA, OLAc, or a combination of the two, revealed that H_2_O was indeed formed under the reaction conditions used for the synthesis of DOA-based InAs QDs. Interestingly, the water released from the reaction did not result in the formation of any surface In or As oxides on the DOA-based InAs QDs (as revealed by both XPS and XRD). Instead, it led to the formation of trimethylsilanol (Me_3_SiOH), which reacted with itself to generate hexamethyldisiloxane ((Me_3_Si)_2_O) and with OLAc to form the corresponding ester, tris­(trimethylsilyl) oleate (Me_3_Si-Oleate). As a result, we identified free Me_3_Si-Oleate and bound Me_3_Si-based moieties as Si-based impurities in the NMR spectrum of DOA-based QDs.

Our work thus demonstrates that further improvements in the quality of (Me_3_Si)_3_As-based InAs QDs can be pursued by employing tertiary alkylamines, which replace primary or secondary ones in their synthesis.

## Experimental Section

### Chemicals

Indium­(III) acetate (InOAc, 99.99%), Oleic acid (OLAc), 1-octadecene (90%), dioctylamine (DOA, 98%), tri-n-octylamine (TOA, 98%), toluene (anhydrous, 99.8%), hexane (anhydrous, 99%), butanol (anhydrous, 99.8%), toluene-d8 (99.6 atom % D). The above chemicals were purchased from Sigma-Aldrich and used without further purification. Tris­(trimethylsilyl)­arsine ((Me_3_Si)_3_As, 99%) was purchased from Dock chemicals and used without further purification. DOA, TOA, and 1-octadecene were degassed at 120 °C under vacuum for 1h before use.

### Synthesis of InAs QDs Using DOA

The synthesis method is based on the study previously reported by Song et al., with some modifications.^30,31^ The synthesis of InAs QDs is carried out in three steps: first, starting InAs QDs are prepared, which are then grown larger through two subsequent steps involving the continuous injection of InAs clusters.

#### First Step

We first prepared indium oleate by mixing 580 mg (2 mmol) of indium acetate, 2 mL of oleic acid (6 mmol) and 3 mL of 1-octadecene in a 100 mL round-bottom flask. The mixture was degassed at 120 °C for 2 h under vacuum. The As precursor solution was prepared inside an argon filled glovebox by mixing 140 μL of ((Me_3_Si)_3_As) (0.5 mmol) with 450 μL of DOA (1.5 mmol) and 1 mL of 1-octadecene in a 4 mL glass vial at room temperature for ∼5 min. This solution was rapidly injected into the reaction flask, which was previously heated up to 280 °C under nitrogen. The InAs QDs were allowed to grow for 10 min after the injection.

#### Second Step

We prepared a dispersion of DOA-based InAs clusters by mixing an indium oleate solution (prepared by degassing at 120 °C under vacuum for 2 h a mixture of 870 mg, 3 mmol, of indium acetate, 2.35 mL of oleic acid, 9 mmol, and 15 mL of 1-octadecene in a 100 mL round-bottom flask) with an As-precursor solution (prepared inside a glovebox by mixing 215 μL of ((Me_3_Si)_3_As), 0.75 mmol, 1.375 mL of DOA, 4.5 mmol, and 3 mL of 1-octadecene in a 10 mL glass vial at room temperature for ∼5 min).

InAs QDs were grown larger by the continuous injection of the DOA-based InAs cluster dispersion into the crude reaction solution of InAs QDs heated up to 280 °C. The injection rate was 8 mL/h for the first hour, followed by a 4 mL/h rate until all the InAs cluster dispersion was completely injected (this step lasted ∼3 h). The reaction was quenched by cooling it to room temperature, and the resulting QDs were purified by adding 15 mL of hexane and 60 mL of butanol, followed by centrifugation at 8000 rpm for 5 min. The precipitate was discarded. Next, 60 mL of butanol was added to the supernatant and the resulting mixture was centrifuged again at 8000 rpm for 5 min. The precipitate was collected and redispersed in 10 mL of hexane, then precipitated again by adding 40 mL of butanol. This cleaning step was repeated twice. Finally, the precipitate was redispersed in 3 mL of hexane. All purification steps were carried out in a glovebox under an argon atmosphere.

#### Third Step

A second DOA-based InAs clusters dispersion was prepared as described in the *second step*. The InAs QDs prepared in the *second step* were mixed with 3 mL of 1-octadecene in a 100 mL flask and degassed at 120 °C for 1 h under vacuum to remove the hexane, leaving the QDs in the 1-octadecene solution. The temperature of the flask was then increased to 280 °C under nitrogen, and the dispersion of DOA-based InAs clusters was injected continuously using a syringe pump. The injection rate was varied over time: for the first 2 h, the injection rate was 2 mL/h, followed by a 1 mL/h rate until all the InAs clusters were injected (the overall injection process lasted ∼20 h). The reaction was then stopped and the flask was allowed to cool to room temperature. The resulting QDs were purified similarly to the method used in the *second step*. The washed final InAs QDs were dispersed in a nonpolar solvent such as octane, toluene, or hexane for further processing.

### Synthesis of InAs QDs Using TOA

The synthesis of InAs QDs using TOA followed the same procedure as that used for DOA, with the only modification being the substitution of DOA with TOA in equivalent molar amounts. Specifically, 656 μL of TOA (1.5 mmol) was used for the synthesis of InAs starting QDs, and 1.96 mL (4.5 mmol) for the preparation of the TOA-based InAs cluster solution. The cleaning procedures were also analogous to those employed for DOA-based InAs QDs.

### Optical Measurements

The absorption spectra were recorded using a Varian Cary 5000 UV–vis–NIR spectrophotometer. The samples were prepared by diluting the QDs solution in 3 mL of tetrachloroethylene in 1 cm path length quartz cuvettes, sealed with airtight screw caps, inside a N_2_ filled glovebox. Aliquots were taken directly from the reaction mixture using a glass syringe, diluted with toluene, and their absorption was measured under ambient conditions.

### X-ray Diffraction (XRD)

XRD patterns were collected using a PANanalytical Empyrean X-ray diffractometer equipped with a 1.8 kW Cu Kα ceramic X-ray tube and a PIXcel3D 2 × 2 area detector, operating at 45 kV and 40 mA. For XRD measurements, specimens were prepared by depositing a concentrated QDs solution onto a silicon zero-diffraction single crystal substrate. Diffraction patterns were recorded under ambient conditions utilizing parallel beam geometry and symmetric reflection mode. The XRD data were analyzed using PANalytical’s HighScore 4.1 software.

### X-ray Photoelectron Spectroscopy (XPS)

Specimens for XPS were prepared in a glovebox by drop casting a few microliters of a concentrated QD solution onto freshly cleaved highly oriented pyrolytic graphite (HOPG, ZYB grade). The specimens were then transferred to the vacuum chamber, avoiding air exposure through an *ad hoc* transfer vessel, to reduce air-induced oxidation. The XPS analyses were carried out with a Kratos Axis Ultra^DLD^ spectrometer, using a monochromatic Al Kα source operated at 20 mA and 15 kV. Wide scans were carried out at a pass energy of 160 eV over an analysis area of 300 × 700 μm^2^. High-resolution analyses were carried out at a pass energy of 20 eV. The Kratos charge neutralizer system was used on all specimens. Spectra were charge corrected to the main line of the carbon 1s spectrum (C–C bonds) set to 284.8 eV. Spectra were analyzed using CasaXPS software (version 2.3.24).^52^

### Bright Field Transmission Electron Microscopy (BF-TEM)

Diluted QDs solutions were drop-cast onto copper TEM grids with an ultrathin carbon film. Overview bright-field TEM images were acquired on a JEOL JEM-1400Plus microscope with a thermionic gun (LaB_6_) operated at an acceleration voltage of 120 kV.

### Thermogravimetric Analysis (TGA)

Thermal gravimetric analysis (TGA) was conducted with a TA Instruments Q500 thermal analyzer under a 50 mL/min nitrogen flux on ∼5 mg of nanocrystals powders. The samples were analyzed after a 5 min equilibration at 30 °C, and then the temperature was increased by a 10 °C/min ramp up to 700 °C.

### Nuclear Magnetic Resonance (NMR)

All NMR experiments were acquired on a Bruker (Bruker, Rheinstetten, Germany) 600 MHz spectrometer fitted with a 5 mm QCI cryoprobe, at 298 K. The lock, matching and tuning, shimming, and the 90° pulse optimization,^53^ were performed automatically on each sample tube, by using Bruker’s routines. The NMR samples were prepared by drying under vacuum a 30 μL aliquot of each InAs QD dispersion. After drying, 1.5 mL of toluene-d8 was added to the QDs to redisperse them. A 600 μL portion of this solution was then used for NMR analysis.

^1^H NMR 32 scans were accumulated, without steady ones and spinning, at a fixed receiver gain (4.5), with 65536 digit points and an interpulse delay of 30 s, over a spectral width of 20.83 ppm with the offset positioned at 6.18 ppm. A smoothing exponential function equivalent to 0.3 Hz was applied to FID (Free Induction Decay) before the Fourier transform.

#### ^13^C NMR {^1^H decoupled}

7618 transients were accumulated after 4 dummy scans, with 32768 digit points, a relaxation delay of 2 s, over a spectral width of 236.65 ppm with offset centered at 100.00 ppm.

^1^H–^13^C HSQC (*Heteronuclear Single-Quantum Correlation*) experiment (edited version) was performed with 64 scans, 1024 digit points, 256 increments and ^1^J_CH_ of 145 Hz, over a spectral width of 15.15 ppm for ^1^H and 165.0 ppm for ^13^C, with a transmitter frequency offset positioned at 7.0 and 75 ppm, respectively. ^1^H–^13^C *HSQC sensitive improved* experiment was run with the same parameters except for the spectral width of 13.02 ppm for ^1^H and 165.0 ppm for ^13^C, with a transmitter frequency offset positioned at 4.7 and 70 ppm, respectively. The ^1^H−^13^C HMBC experiment was recorded by using 16 FIDs, 4096 digit points, 128 increments, and ^1^ *J*CH long range of 10 Hz, a spectral width of 15.15 ppm for 1H and 220.00 ppm for ^13^C, with a transmitter frequency offset at 7.00 and 100.00 ppm, respectively.

## Results and Discussion

InAs QDs were synthesized following the procedure reported by Song et al. with several modifications.^30^ In detail, to prepare the starting InAs QDs, DOA was mixed with (Me_3_Si)_3_As and 1-octadecene, and the resulting solution was injected in a reaction flask containing In-oleate and 1-octadecene at 280 °C (see the Experimental section). InAs QDs having an excitonic absorption peak at ∼755 nm with a HWHM of 97.2 meV and a P/V ratio of 1.36 were prepared by quenching the reaction after 10 min (Figure 1a-c, time = 0 h). The QDs were then grown larger by a continuous injection of DOA-based InAs clusters, which was performed at 280 °C and lasted ∼3.5 h, eventually shifting the first excitonic absorption peak to 937 nm, with a HWHM of 64 meV and a P/V ratio of 2.4 (Figure 1a-c, time = 3.5 h). After cleaning the QDs, these were grown larger by a second seeded growth step performed at 280 °C for 20 h. The final InAs QDs featured an absorption peak at 1045 nm with a HWHM of 53 meV and P/V ratio of 2.2 (Figure 1a-c, time = 23.5 h). Such low HWHM and high P/V ratios indicate excellent control over size distribution of the InAs QDs. This was primarily achieved by employing DOA-based InAs clusters prepared with a DOA:(Me_3_Si)_3_As ratio of 6:1, (different from the 3:1 ratio used by Song et al.), and by finely tuning the injection rate of the InAs clusters (see Experimental section in the SI for details).^31,51^ The XRD pattern of these QDs was consistent with cubic zinc-blende bulk InAs (ICSD 98–002–4518) with no presence of secondary phases (Figure 1d). Notably, in contrast to previous reports,^30,51^ no indium nor arsenic oxide peaks were detected in the XRD pattern of the DOA-based InAs QDs. According to transmission electron microscopy (TEM) analysis, the final sample consisted of faceted particles with a homogeneous size distribution of 5.7 ± 0.5 nm (Figure 1e and Figure S1a,c).

**Figure 1:**
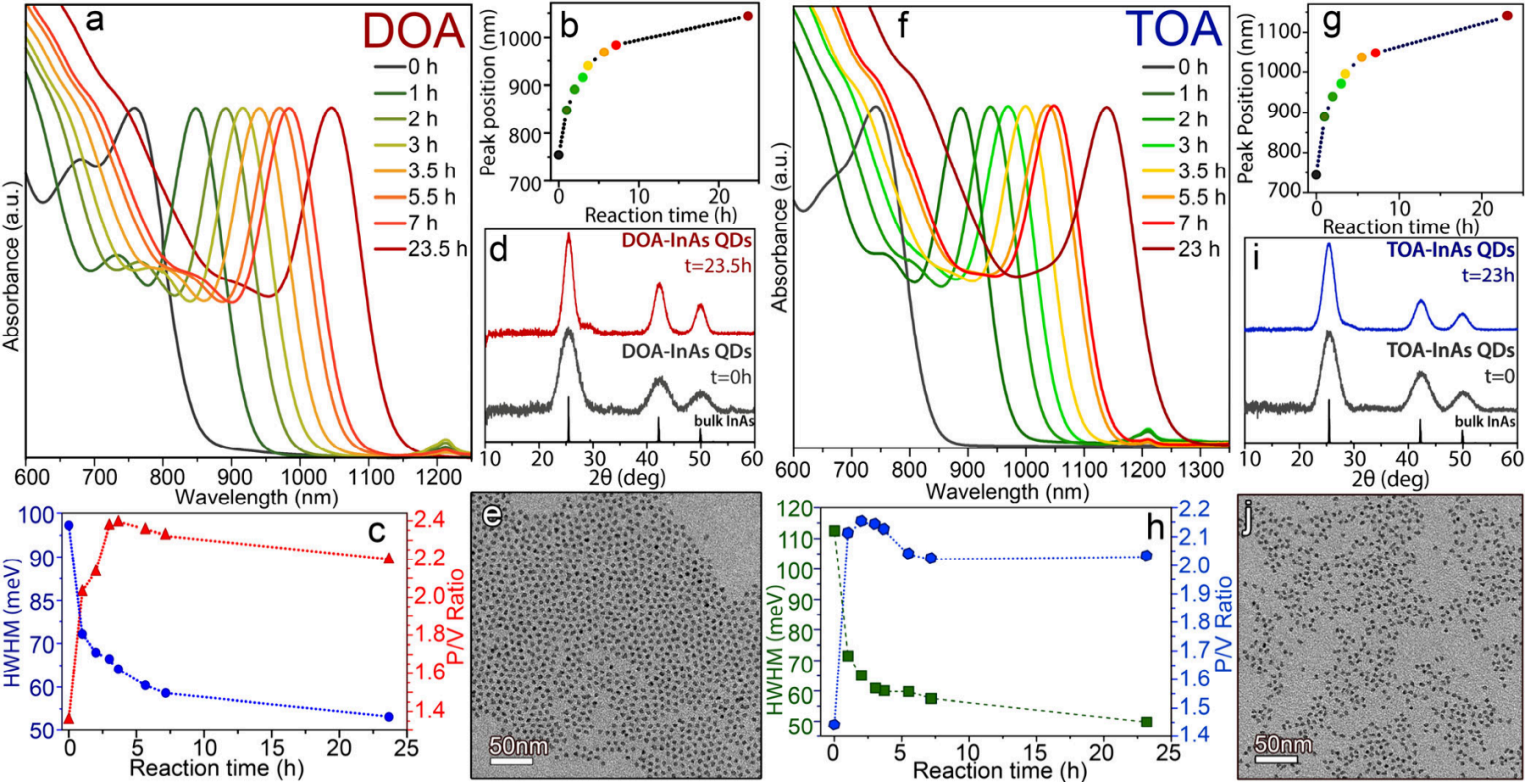
(a) Evolution of the optical absorption spectra of InAs QDs synthesized with (a) DOA or (f) TOA at different reaction times along with the corresponding (b, g) excitonic peak positions and (c, h) HWHM and P/V ratios. The reaction time 0 corresponds to the time at which the seeded growth starts. XRD patterns of InAs QDs prepared with (d) DOA or (i) TOA at different reaction times. (e) BF-TEM image of the final InAs QDs prepared with (e) DOA (reaction time = 23.5 h) or (j) TOA (reaction time = 23 h).

We then proceeded with the synthesis of InAs QDs using TOA. To ensure a clear comparison between the products of the two procedures, we kept all reaction parameters constant, with the only difference being the replacement of DOA with TOA. TOA, like DOA, was seen to reduce the reactivity of (Me_3_Si)_3_As, resulting in InAs QDs with size and absorption features that were similar to those of QDs prepared with DOA (Figure 1f).^31,51^ Indeed, after a reaction time of 10 min the InAs QDs prepared with TOA featured a first excitonic absorption peak at ∼750 nm with an HWHM of 114 meV and a P/V ratio of 1.44 (Figure 1f-h, time = 0 h). Such QDs were then grown larger by a continuous injection of TOA-based InAs clusters following the same procedure reported for the DOA case. After the first seeded growth step, performed at 280 °C for 3h, we obtained InAs QDs featuring an absorption peak at 972 nm (HWHM of 61 meV and a P/V ratio of 2.14) (Figure 1f-h, time 3h). The second seeded growth step (performed at 280 °C for 20 h) led to InAs QDs with an excitonic absorption peak located at 1140 nm and having a HWHM of 55 meV and a P/V ratio of 2.03. Here, as well, the XRD pattern of the QDs was consistent with cubic zinc-blende bulk InAs (ICSD 98-002-4518) with no presence of secondary phases (Figure 1i). Similarly to the DOA case, TEM analysis of the TOA-based QDs indicated that they were faceted nanocrystals with an average size of 6.0 ± 0.6 nm (Figure 1j and Figure S1b,d).

The comparison between DOA- and TOA-based InAs QDs indicated that both amines provided excellent control over the nucleation and growth of InAs QDs, resulting in (i) high P/V ratios (∼2) and remarkably narrow HWHM (∼50 meV) which are record values for these QDs (Table S1); (ii) no presence of secondary crystal phases or secondary QD populations. However, important differences were observed during the purification of the resulting QD products. QDs synthesized with DOA were particularly difficult to clean, as they often formed gel-like agglomerates during the different washing steps (Figure S2). This could lead to the loss of the entire QD batch when excessive amounts of antisolvent were added, causing complete precipitation of the product (Figure S3 for details). Moreover, even when the washing cycles led to a final stable colloidal dispersion, the resulting DOA-based QDs remained visibly contaminated with organic residues, exhibiting a “sticky” texture upon drop-casting and poor electrical conductivity (Figures S4 and S5). In contrast, TOA-based QDs were significantly easier to purify, with the organic byproducts being easily removed without particular precautions, yielding cleaner and more uniform films upon drop-casting and higher electrical conductivity compared to the DOA-based QDs (Figures S4 and S5).

To investigate structural and compositional differences between the two InAs QD samples, we performed TGA and XPS analyses on the final products from the two seeded growth approaches (i.e., QDs absorbing at 1045 nm in the DOA case, and QDs absorbing at 1140 nm in the TOA case). Consistent with the qualitative observations discussed in the previous paragraph, the TGA analyses revealed that the DOA-based QDs contained a significantly higher proportion of organic/metal–organic species compared to the TOA-based QDs (Figure 2a-b). For the DOA-based InAs QDs, we observed an initial weight loss of around 5% at 134 °C, followed by a further 41% loss at 373 °C (Figure 2a), in line with previous reports on analogous systems.^51^ In contrast, the TOA-based QDs exhibited only a 29% weight loss at approximately 380 °C (Figure 2b).

**Figure 2:**
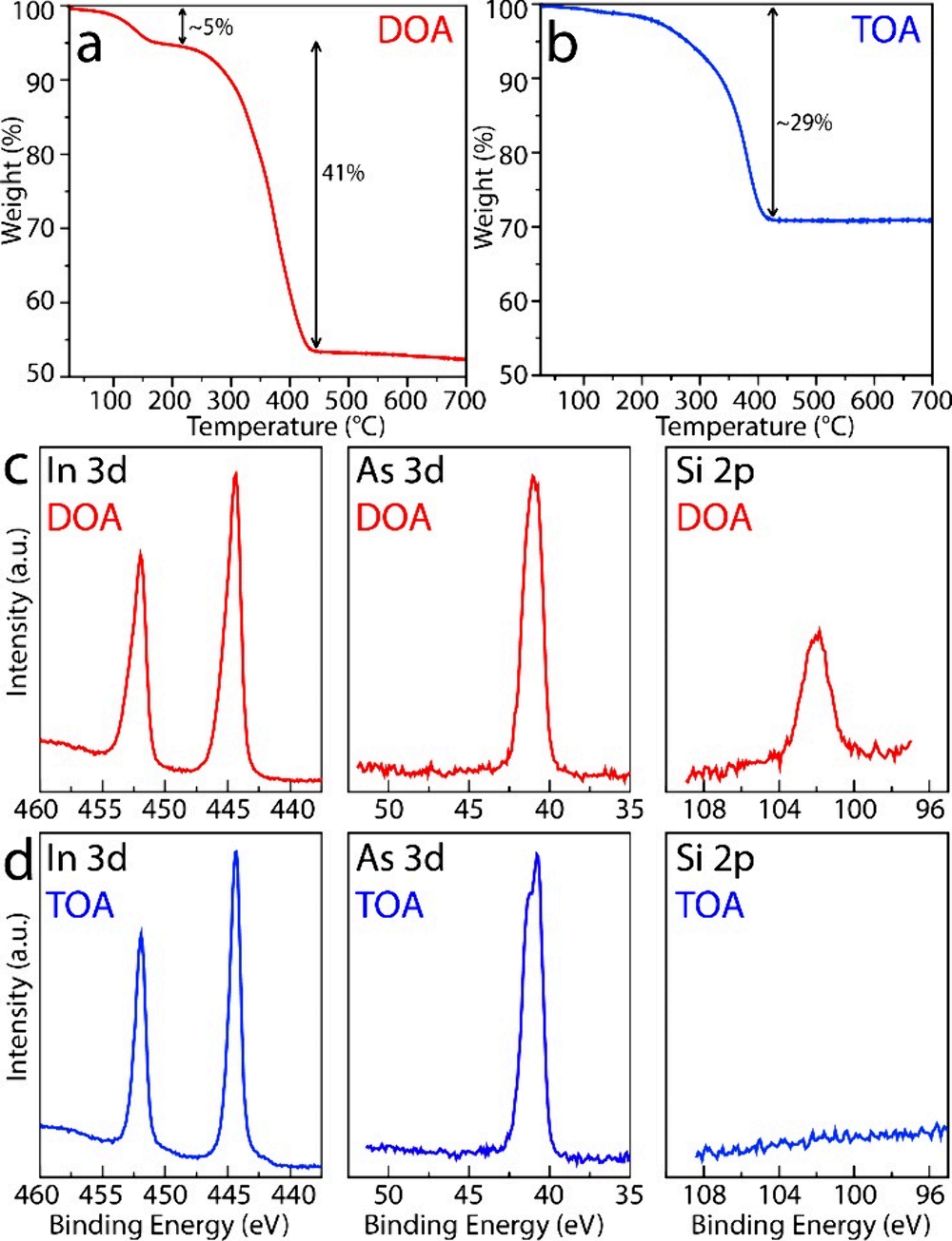
TGA curves of (a) DOA- and (b) TOA-based InAs QDs. In 3d, As 3d and Si 2p XPS spectra acquired for (c) DOA- and (d) TOA-based InAs QDs.

XPS analysis of the two samples revealed no presence of As oxides on the surface of these QDs, contrary to what has been typically reported in the literature to date for DOA-based InAs QDs.^30,51^ In both DOA- and TOA-QD samples the As 3d peak was centered at ∼41 eV, in agreement with previous reports on arsenides,^46,54,55^ with no signal observed in the 42–46 eV binding energy range characteristic of As_2_O_3_ (Figure 2c-d, middle panels). The In 3d signals displayed the main In 3d_5/2_ component centered at 444.3 ± 0.2 eV (Figure 2c-d, left panels), consistent with the presence of InAs.^54^ The spectrum collected for the DOA-QD sample also exhibited a noticeable broadening on the high binding energy side of the In 3d peaks, which was almost absent in the TOA case. Peak decomposition, shown in Figure S6, revealed the presence of a second In chemical state, with the In 3d_5/2_ component centered at 445.2 ± 0.2 eV. This energy value, along with the O 1s peak at 531.8 ± 0.2 eV (Figure S6), aligns with literature reports on In carboxylates,^56^ which are present in the ligand shell of these QDs. Of particular relevance is the fact that the DOA-based sample exhibited a Si 2p peak at ∼102 eV binding energy, consistent with that of silicone/siloxanes (i.e., organosilicon featuring Si–O bonds),^57^ which was absent in the TOA sample (Figure 2c-d, right panels). The presence of Si-based species in the DOA-based sample was further confirmed by scanning TEM energy dispersive spectroscopy (EDS) compositional mapping, which revealed a distinct Si signal that was homogeneously distributed across the carbon support film and not colocalized with the InAs QDs (Figure S7).

To better elucidate the nature of the Si species, we recorded ^1^H NMR and HSQC spectra of DOA-based InAs QDs, which revealed a sharp peak at 0.25 ppm and a broad peak at 0.70 ppm, compatible with Me_3_Si-based moieties (Figure 3a,b). In particular, based on the phase of the peaks in the edited HSQC spectrum and chemical shift considerations, we attributed the broad signal at 0.70 ppm in ^1^H and −2.58 ppm in ^13^C to a CH_3_ moiety belonging to Me_3_Si-based species bound to the surface of the QDs (Figure 3b). To identify the nature of such species, elucidate their formation, and understand the possible side reactions occurring during InAs QDs synthesis, we performed a series of control experiments in which we analyzed the corresponding products by ^1^H and ^13^C NMR. These control experiments involved the reaction of (Me_3_Si)_3_As or Me_3_SiOH with either DOA, OLAc, or combinations of DOA + OLAc or TOA + OLAc at elevated temperatures (i.e., 250 °C). Me_3_SiOH was selected since it is a reaction product of Me_3_Si-containg species with H_2_O, which is, in turn, produced by the condensation of DOA and OLAc (see below). The products of the reactions were identified by spiking with authentic compounds, commercially available whenever possible, or freshly synthesized, whose structures were completely characterized and assigned by NMR (Figures S8–S32). Our results indicated that

- (Me_3_Si)_3_As reacts with OLAc to form the corresponding Me_3_Si-Oleate (eq 1 and Figures S18–S19);
1

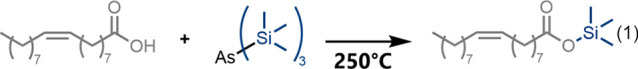

- Me_3_SiOH reacts with OLAc to form the corresponding Me_3_Si-Oleate and marginally with itself, leading to (Me_3_Si)_2_O (eq 2, Figure 3c and Figures S20–S21);
2

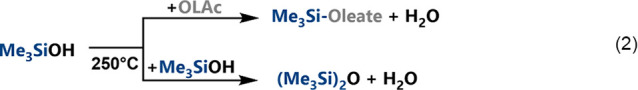

- Neither Me_3_SiOH nor (Me_3_Si)_3_As react with DOA to form the corresponding amide, namely dioctyltrimethylsilylamine (Figures S22–S25);
- Of particular interest was the reaction of (Me_3_Si)_3_As with a mixture of DOA and OLAc, which resulted in a significant formation of various products including the N,N-dioctyl-(9Z)-octadec-9-enamide (eq 3), Me_3_Si-Oleate, Me_3_SiOH and (Me_3_Si)_2_O (Figure 3d and Figures S26–S29).
- The reaction of (Me_3_Si)_3_As with a mixture of TOA and OLAc resulted mostly in Me_3_Si-Oleate (Figures S30–S32).

**Figure 3:**
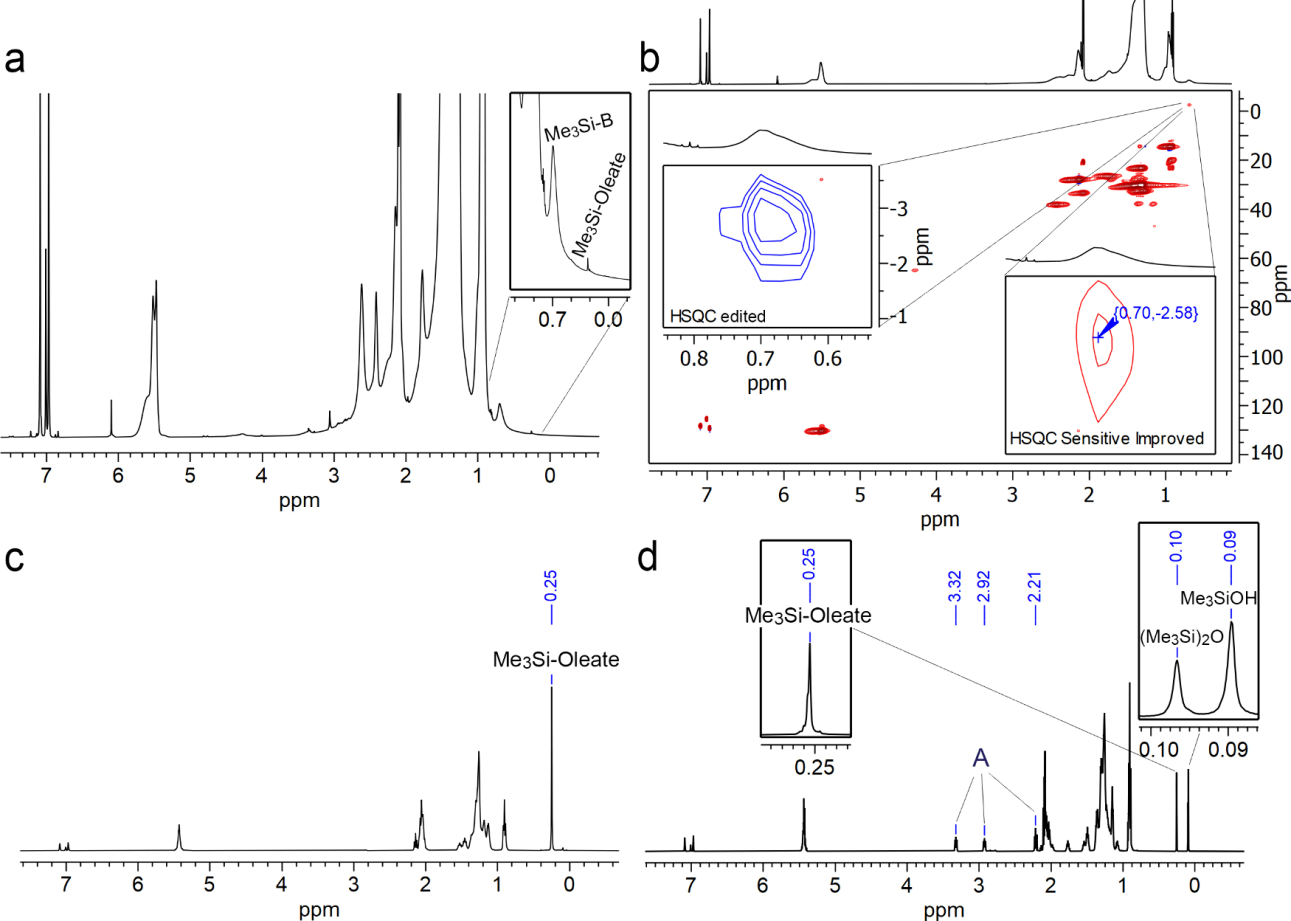
(a) ^1^H NMR and (b) ^1^H–^13^C HSQC NMR spectra of DOA-based InAs QDs in toluene-d8. The insets show the edited version (left inset), in which CH_3_ groups are represented in blue, and the sensitive improved (right inset). (c–d) ^1^H NMR spectra in toluene-d_8_ of the reactions performed at 250 °C: (c) Me_3_SiOH + OLAc; (d) (Me_3_Si)_3_As + DOA + OLAc. A= N,N-dioctyl-(9Z)-octadec-9-enamide.

Overall, our control experiments indicated the following:

i) DOA and OLAc condense even at temperatures that are lower than the one employed for the InAs QDs synthesis (i.e., 250 °C vs 280 °C), releasing H_2_O (eq 3);
3

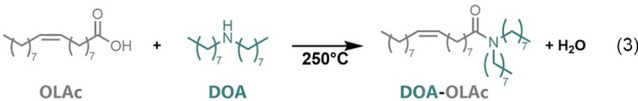

ii) Notably, no ketones were observed in our control experiements (Figures S8–S32), excluding ketonization of OLAc as a source of water, as previously hypotesized by different groups;^51^
iii) the water that is formed can subsequently react with Me_3_Si-containing species, promoting the generation of significant amounts of Me_3_SiOH. Me_3_SiOH can then further react with itself and with OLAc, resulting in the formation of (Me_3_Si)_2_O, Me_3_Si-Oleate, and additional water, in a cascade of reactions (eq 2);^58^
iv) the sharp peak at 0.25 ppm present in the ^1^H NMR spectrum of DOA-InAs QDs could be identified as free Me_3_Si-Oleate (Figure 3a);
v) the broad peak at 0.69 ppm in the ^1^H NMR spectrum of DOA-InAs QDs (named Me_3_Si–B in Figure 3a) was attributed by HSQC to Me_3_Si-based species bound to the QDs’ surface (Figure 3b); however, its precise identification was not possible.

These results could be rationalized considering that, under the specific reaction conditions used for the synthesis of DOA-InAs QDs, the water generated in situ by the condensation of DOA with OLAc led to the substantial formation of Amide, Me_3_Si-Oleate, and (Me_3_Si)_2_O species, which are difficult to remove during the QDs cleaning steps (thus leading to the experimentally observed washing problems, as mentioned previously). In contrast, for TOA-InAs QDs, the formation of Me_3_Si-Oleate is limited, likely because (Me_3_Si)_3_As is consumed to form InAs QDs rather than Me3Si-oleate, and the residual Me_3_Si- species are not hydrolyzed by water during the reaction and therefore cannot react with OLAc to form Me_3_Si-Oleate. It is important to note that, under slightly different reaction conditions employed by other groups in the synthesis of DOA-based InAs QDs, surface oxides have been observed by those groups, indicating that the fate of the in situ generated H_2_O is not straightforward.^30,51^

## Conclusion

In conclusion, we have demonstrated that trioctylamine (TOA), similarly to dioctylamine (DOA), can reduce the reactivity of (Me_3_Si)_3_As, enabling fine-tuning of InAs QDs size while maintaining a good size distribution. Specifically, our reaction conditions allowed to tune the excitonic absorption peak of InAs QDs up to 1100 nm with a HWHM as low as ∼ 50 meV and a P/V ratio around 2, the latter being a record value for these QDs. Moreover, we have shown that TOA, unlike DOA, prevents the in situ formation of water, which is caused by the condensation reaction between DOA and oleic acid (OLAc) at the high temperatures used in QD synthesis (i.e., 280 °C). This in situ generated water was found to lead to the formation of (Me_3_Si)_2_O and Me_3_Si-Oleate species, which contaminate the DOA-based InAs QDs. As a consequence of this contamination, the purification of DOA-based InAs QDs proved challenging, often resulting in loss of the final QD product. Even when properly washed, the QDs still contained both Me_3_Si-Oleate and surface-bound Me_3_Si-based species. We envisage that adjusting the bulkiness of the tertiary amines (i.e., by altering the alkyl groups attached to nitrogen atom) can be explored to further tune reaction kinetics, potentially improving control over the size and size distribution of the InAs QDs.

## Supplementary Material



## Notes

Size distribution plots, HRSTEM, Experimental observations during washing of QDs, FET tranfer properties, XPS analysis, STEM-EDS analysis, NMR analysis. (PDF)
