# Supplementary material for: Trioctylamine in the Synthesis of Tris(trimethylsilyl)arsine-Based InAs Quantum Dots Prevents the Formation of Si-Based Byproducts
Source: J Am Chem Soc. 2025 Oct 21;147(44):40389–97. doi: 10.1021/jacs.5c11775 (PMC12593344; doi:10.1021/jacs.5c11775)
Supplement: Supplementary file 1 [file ja5c11775_si_001.pdf]

Supporting Information

Tri-Octylamine in the Synthesis of Tris(Trimethylsilyl)Arsine-based InAs Quantum Dots Prevents the Formation of Si-based Byproducts

Satyaprakash Panda[a,b], Lutfan Sinatra[c], Khursand E. Yorov[c], Galih R. Suwito[c], Alexander Bessonov[c], Marat Lutfullin[c], Luca Goldoni[d], Enrico Bergamaschi[e], Rosaria Brescia[f], Mirko Prato[d], Giorgio Divitini,[g] Luca De Trizio*[h] and Liberato Manna*[a]

[a] Nanochemistry, [d] Materials Characterization, [e] Molecular Modelling & Drug Discovery, [f] Electron Microscopy and [g] Electron Spectroscopy and Nanoscopy, [h] Chemistry Facility, Istituto Italiano di Tecnologia, Via Morego 30, 16163 Genova, Italy

[b] Dipartimento di Chimica e Chimica Industriale, Università di Genova, 16146 Genova, Italy

[c] Quantum Solutions, Unit 8-Innovation Quarter, Oxford Technology Park, Kidlington OX5 1GN, Oxford-UK (www.quantum-solutions.com)

TEM analysis

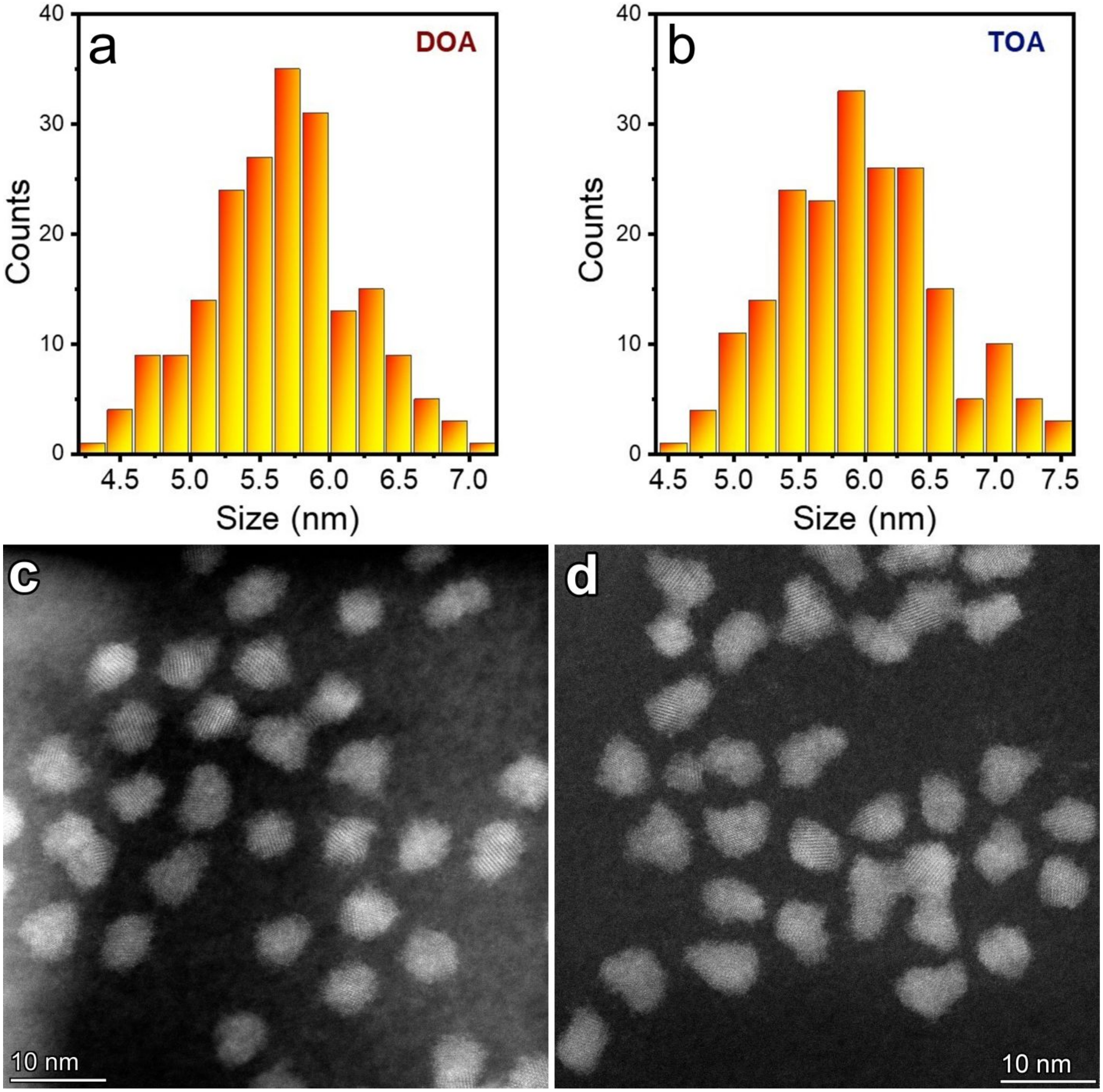

Figure S1. Size distribution histograms obtained from bright field TEM images of InAs QDs synthesized using (a) DOA or (b) TOA. High-resolution scanning transmission electron microscopy (STEM) micrographs of (c) DOA-based and (d) TOA-based InAs QDs. The size of the QDs was measured using ImageJ software.[1]

High resolution scanning transmission electron microscopy images were acquired at an acceleration voltage of 300 kV in a Thermo Fisher Spectra 300 S/TEM, acquiring the high-angle annular dark field (HAADF) signal. The current was set to 200 pA and the convergence angle to 25 mrad. Samples were dispersed on ultra-thin carbon films for analysis.

Table S1. Excitonic peak position, HWHM and P/V ratios of InAs QD samples reported in the present work and in literature

Precursor system	Excitonic peak position (nm)	HWHM (meV)	P/V ratio	
$(Me_3Si)_3As$ + DOA	1045	53	2.2	This work
$(Me_3Si)_3As$ + TOA	1140	55	2.03	This work
$(Me_3Si)_3As$ + DOA	~1140	55	1.3	Song et. al.[2]
$(Me_3Si)_3As$	1200	92	---	Franke et. al.[3]
$(Me_3Si)_3As$	1100	~70	---	Tamang et. al.[4]

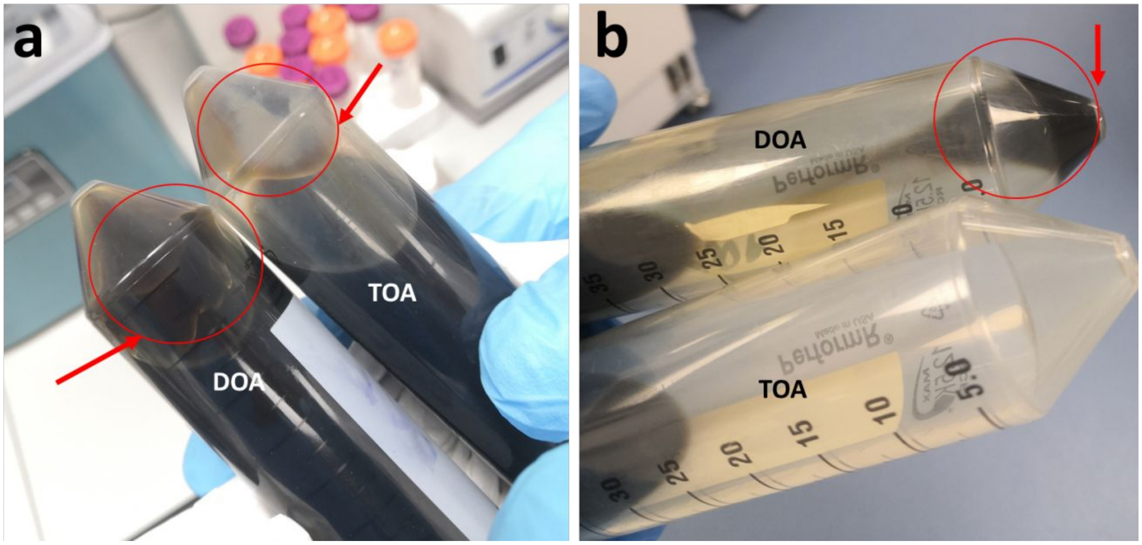

Figure S2. Photographs of the InAs QD products obtained using either DOA or TOA are shown after a) addition of the antisolvent (butanol) to the crude reaction solution and b) upon the first washing step and redispersion in hexane. In a), it is clearly visible that DOA-based QDs are accompanied by a more abundant amount of organic, gel-like byproducts compared to the TOA-based sample. In b), the contrast becomes more evident: while the TOA-based QDs could be fully redispersed in hexane, showing no signs of precipitated byproducts, a portion of the DOA-based sample remained trapped in a sticky, gel-like precipitate that could not be redispersed, indicating incomplete purification and residual contamination.

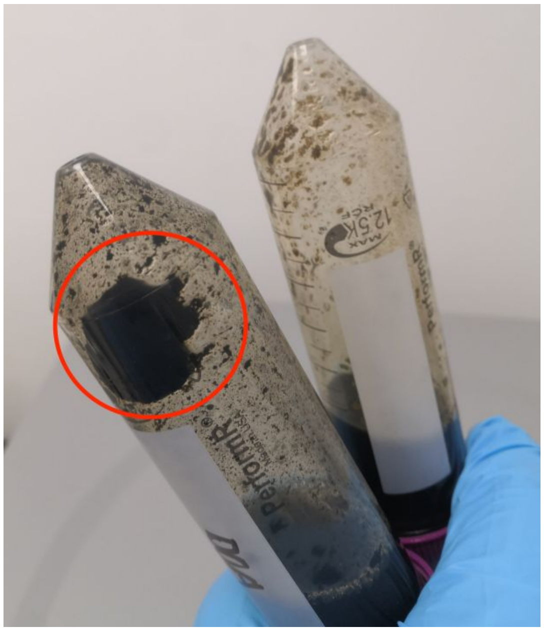

Figure S3. Loss of DOA-based QD product upon improper washing. It is observed that on addition of excessive amount of antisolvent (i.e. butanol), DOA-based InAs tend to precipitate completely, which was then difficult to redisperse.

From the observation of Figure S2 and S3, it is evident that DOA-based InAs QDs pose greater difficulty and complexity in the washing procedure compared to the TOA-based counterparts.

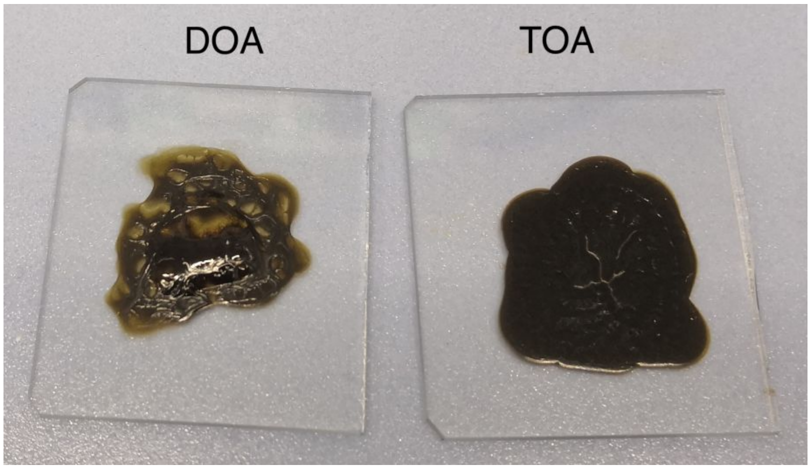

Figure S4. Drop-cast films of (a) DOA-based and (b) TOA-based QDs. The DOA-based QDs form a sticky film indicative of residual organic contamination, while the TOA-based QDs yield a powdery film consistent with a cleaner product.

To investigate the impact of byproducts on the electrical transport properties of InAs QDs, we conducted electrical measurements on films of DOA- and TOA-based QDs deliberately without performing any surface ligand treatment. InAs QD films were deposited on pre-patterned Si substrates with thermal oxide (n++ Si/90 nm SiO_2) and interdigitated Au electrodes. We prepared two device variants: as deposited and baked (with post-heat treatment at 200°C for 30 min) (**Figure S5**). All the FET fabrication steps were carried out inside a glovebox. The FET transfer characteristic measurements were performed using Keithley 4200-SCS Semiconductor Characterization System. The carrier mobility was extracted from the linear regime of the FET transfer curve. It was calculated by fitting the experimental data to the following equation: $\mu = \frac{L}{WC_iV_D}\frac{dI_D}{dV_G}$, where L, W, C_i, V_D, I_D, and V_G are the channel length (i.e., 10 µm), channel width (i.e., 10 mm), capacitance per unit area (i.e., 3.84 × 10^{-4} F.m^{-2}), drain voltage (i.e., 1 V), drain current, and gate voltage, respectively.

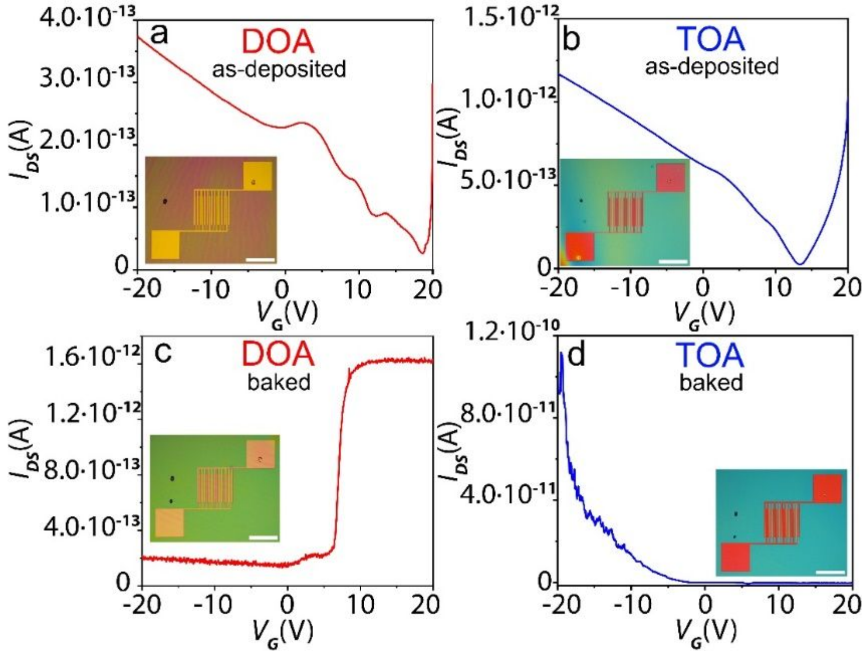

Figure S5. FET transfer characteristics (at 300 K) of InAs QDs samples: (a) DOA and (b) TOA as deposited, (c) DOA and (d) TOA after baking. A plan-view optical image of each FET device is shown for each transfer curve (scale bars are all 0.5 mm).

Table S2. FET data for DOA- and TOA-based InAs QD films

Sample	Transport	Electron mobility (cm²/V·s)	Hole mobility (cm²/V·s)
DOA- QDs, as deposited	Ambipolar	$1.6 \cdot 10^{-9}$	$7.79 \cdot 10^{-10}$
DOA- QDs, baked	Unipolar (electron)	$6.52 \cdot 10^{-9}$	-
TOA- QDs, as deposited	Ambipolar	$3.08 \cdot 10^{-9}$	$2.44 \cdot 10^{-9}$
TOA- QDs, baked	Unipolar (hole)	-	$7.74 \cdot 10^{-8}$

As shown in **Figure S5a, b** and **Table S1**, both as-deposited DOA- and TOA-based InAs QD samples exhibited ambipolar behavior. In contrast, the baking treatment transformed the ambipolar QD films into unipolar films, with electrons as the majority carriers in the DOA-based InAs films and holes as the majority carriers in the TOA-based films (**Figure S5c, d** and **Table S1**). Our electrical characterizations revealed that TOA-based QDs exhibited higher carrier mobility compared to DOA-based QDs. In addition, the FET on-off ratio showed that TOA-based QDs have higher conductivity than DOA-based QDs, with a two-order-of-magnitude difference in the baked samples.

XPS analysis of O 1s, C 1s and In 3d peaks of DOA- and TOA-based InAs QDs

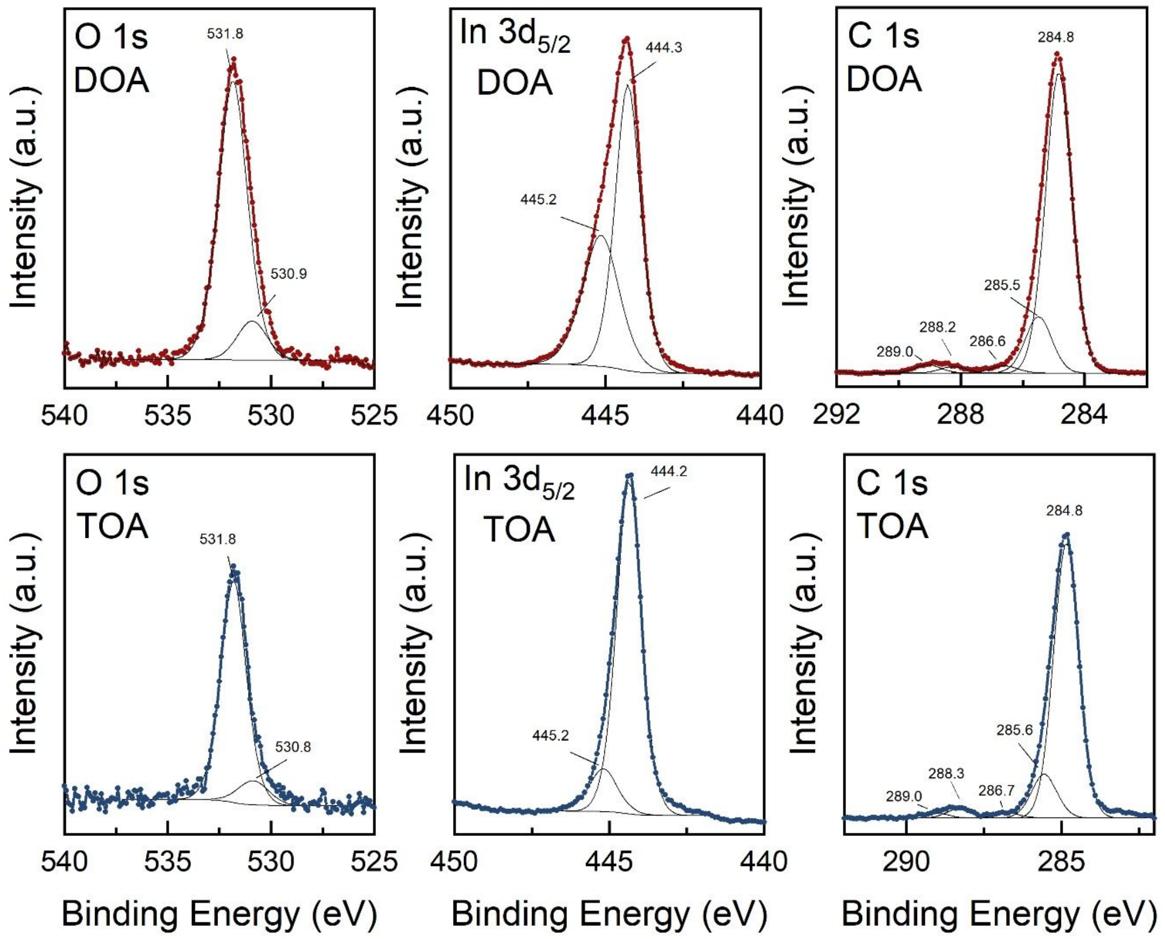

Figure S6. O 1s, In $3d_{5/2}$ and C 1s XPS spectra collected on DOA- (top row) and TOA- (bottom row) QDs samples, shown together with the results of the decomposition process. As discussed in the main text, the O 1s peak at 531.8 ± 0.2 eV, together with the In $3d_{5/2}$ peak at 445.2 ± 0.2 eV, suggests the presence of In carboxylates, which are known to constitute the ligand shell of these QDs. The relative amount of In oleate species is higher in the DOA- sample because of the higher content of organics within the sample, reducing the effectively probed QDs volume via XPS. A second low-intensity oxygen component, centered at approx. 530.8 eV, was also needed to better reproduce the experimental O 1s signal. This is most likely to be attributed to adventitious organic species, as C-OH groups, and not to oxygen in In_2O_3, for which a oxygen signal is expected at 529.5 eV.[5]

STEM-EDS analysis of DOA-based InAs QDs

DOA-based InAs QDs were drop cast onto an ultrathin C/lacey C/Cu grid, previously plasma-treated to remove any residues from film preparation. The analysis was performed employing an image-Cs-corrected JEOL JEM-2200FS TEM, equipped with a Bruker X-Flash 5060 EDS system, operated at 200 kV. The EDS quantification was obtained from the summed spectra acquired from a large number of QDs, using the Cliff-Lorimer method for the Kα peaks of C, Si and As and the Lα peak of In.

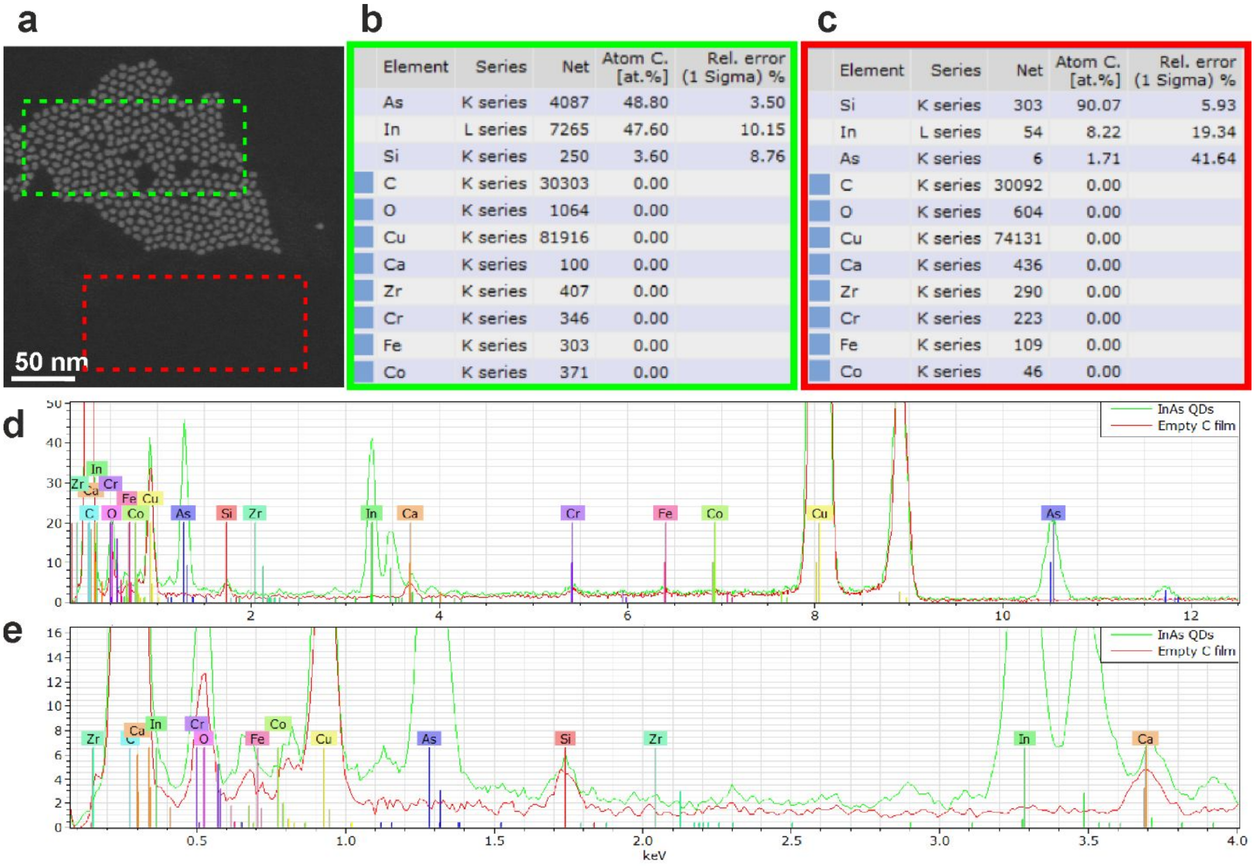

(b)

Element	Series	Net	Atom C. [at.%]	Rel. error (1 Sigma) %
As	K series	4087	48.80	3.50
In	L series	7265	47.60	10.15
Si	K series	250	3.60	8.76
C	K series	30303	0.00	
O	K series	1064	0.00	
Cu	K series	81916	0.00	
Ca	K series	100	0.00	
Zr	K series	407	0.00	
Cr	K series	346	0.00	
Fe	K series	303	0.00	
Co	K series	371	0.00	

(c)

Element	Series	Net	Atom C. [at.%]	Rel. error (1 Sigma) %
Si	K series	303	90.07	5.93
In	L series	54	8.22	19.34
As	K series	6	1.71	41.64
C	K series	30092	0.00	
O	K series	604	0.00	
Cu	K series	74131	0.00	
Ca	K series	436	0.00	
Zr	K series	290	0.00	
Cr	K series	223	0.00	
Fe	K series	109	0.00	
Co	K series	46	0.00	

Figure S7. STEM-EDS quantitative analysis of Si in final InAs QDs synthesized with DOA. (a) High-angle annular dark field (HAADF)-STEM image of QDs and (b) quantification obtained in areas containing a large number of QDs, compared with (c) quantification in neighboring empty regions, with identical area (e.g., see panel (a)), showing no significant difference in net counts for Si (column "Net"). (d) Raw spectra obtained by summing areas (green) with and (red) without QDs, selected as shown in panel (a). The X-rays peaks of Cu, Ca, Zr, Cr and Fe are due to the TEM grid and components of the experimental setup hit by back-scattered electrons.

NMR Characterization

^{13}C NMR {^{1}H decoupled}. 7618 transients were accumulated after 4 dummy scans, with 32768 digit points, a relaxation delay of 2 s, over a spectral width of 236.65 ppm with offset centered at 100.00 ppm.

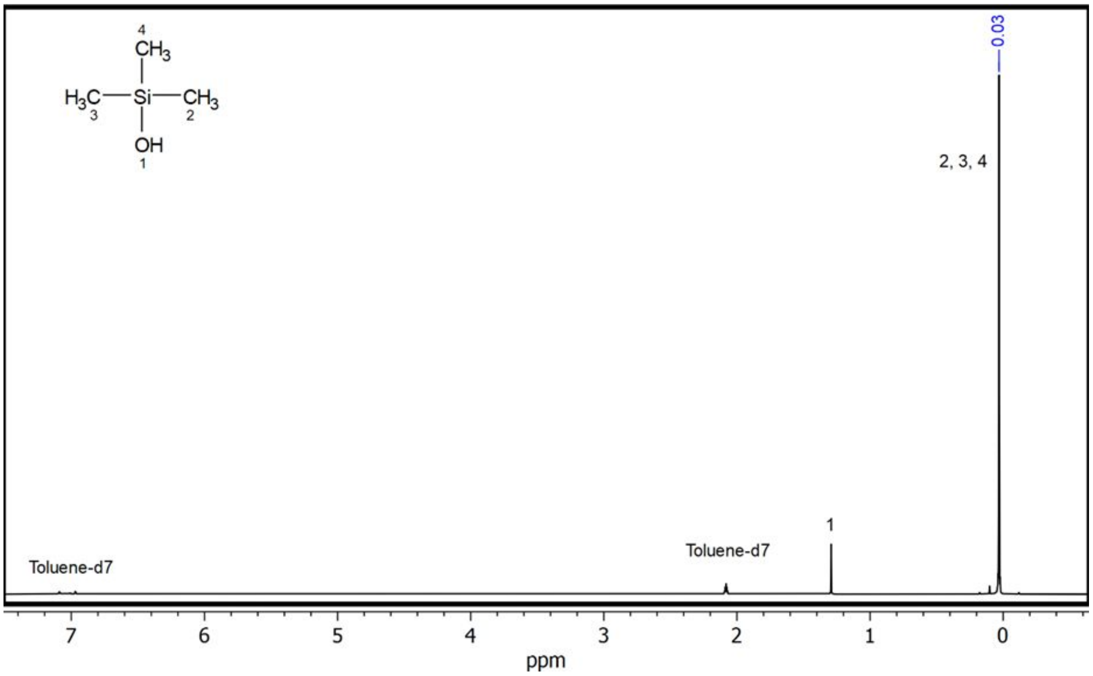

Figure S8. ^{1}H NMR spectrum of Me_3SiOH (Sigma Aldrich, 97.5%) in toluene-d, the structure and peaks assignment are embedded.

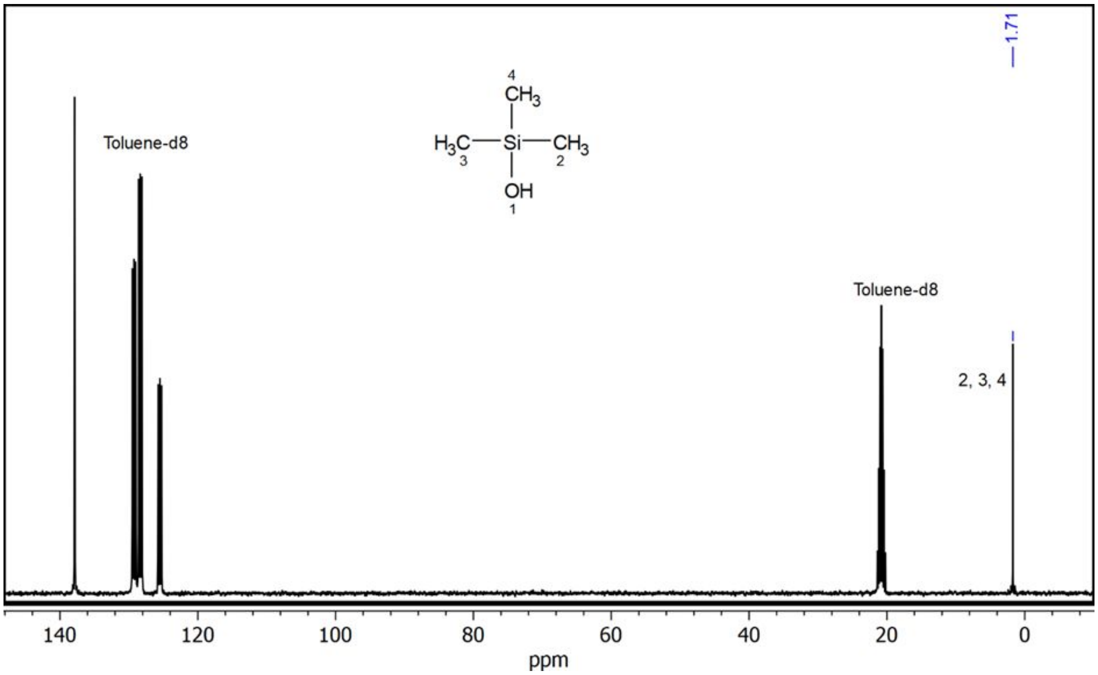

Figure S9. ^{13}C NMR spectrum of Me_3SiOH (Sigma Aldrich, 97.5%) in toluene-d, the structure and peaks assignment are embedded.

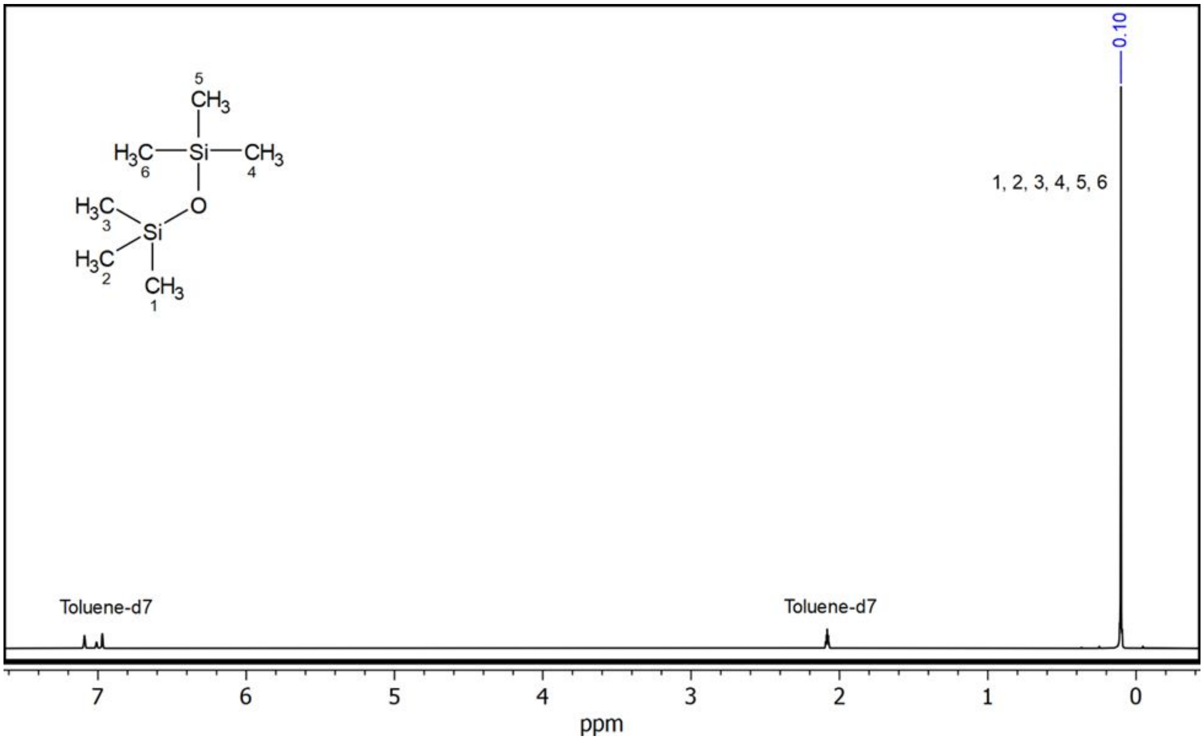

Figure S10. ^{1}H NMR spectrum of $(Me_3Si)_2O$ (Sigma Aldrich, 98%) in toluene-d, the structure and peaks assignment are embedded.

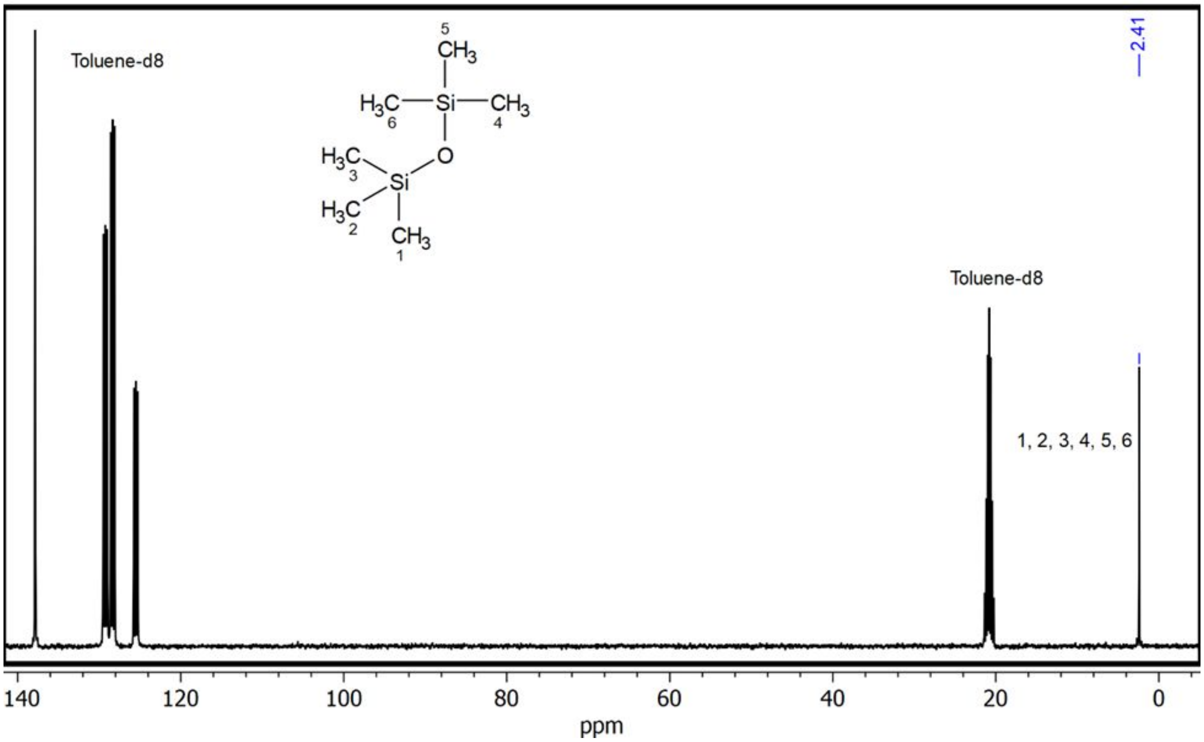

Figure S11. ^{13}C NMR spectrum of $(Me_3Si)_2O$ (Sigma Aldrich, 98%) in toluene-d, the structure and peaks assignment are embedded.

Chemicals

Dry tetrahydrofuran (Sigma Aldrich, 99.9%), dry pyridine (Sigma Aldrich, 99.8%), diisopropylethylamine (Sigma Aldrich, 98.0%), chlorotrimethylsilane (TMS-Cl, Sigma Aldrich, 99.0%), cyclohexane (Sigma Aldrich, 99.5%), dry dichloromethane (Thermo Scientific, 99.9%), triethylamine (Sigma Aldrich, 99.5%).

Procedure for the synthesis of Me_3Si-Oleate

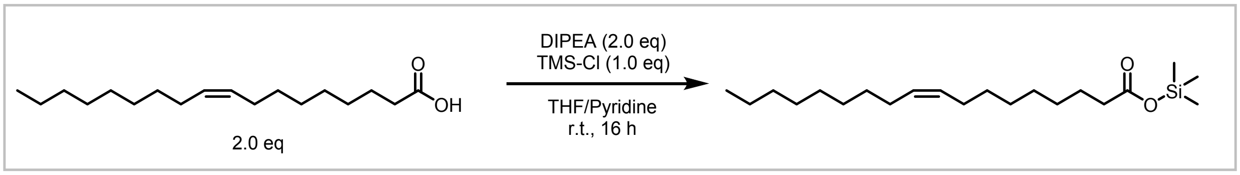

In a screwcap vial with septum, oleic acid (1.0 mmol, 316 µL, 2.0 equivalents) was dissolved in dry tetrahydrofuran (THF, 0.3 ml) and dry pyridine (0.2 ml) under nitrogen atmosphere. To this solution N,N-diisopropylethylamine (DIPEA, 1.0 mmol, 175 µL, 2.0 equivalents) and chlorotrimethylsilane (0.5 mmol, 70 µL, 1.0 equivalents) were subsequently added. The reaction was left stirring overnight at room temperature. Cyclohexane was then added to the reaction mixture and the slurry was filtered. The volatiles were then removed, and the resulting pale-yellow oil was characterized without further purification. The product was stored in a -20°C freezer under nitrogen atmosphere.

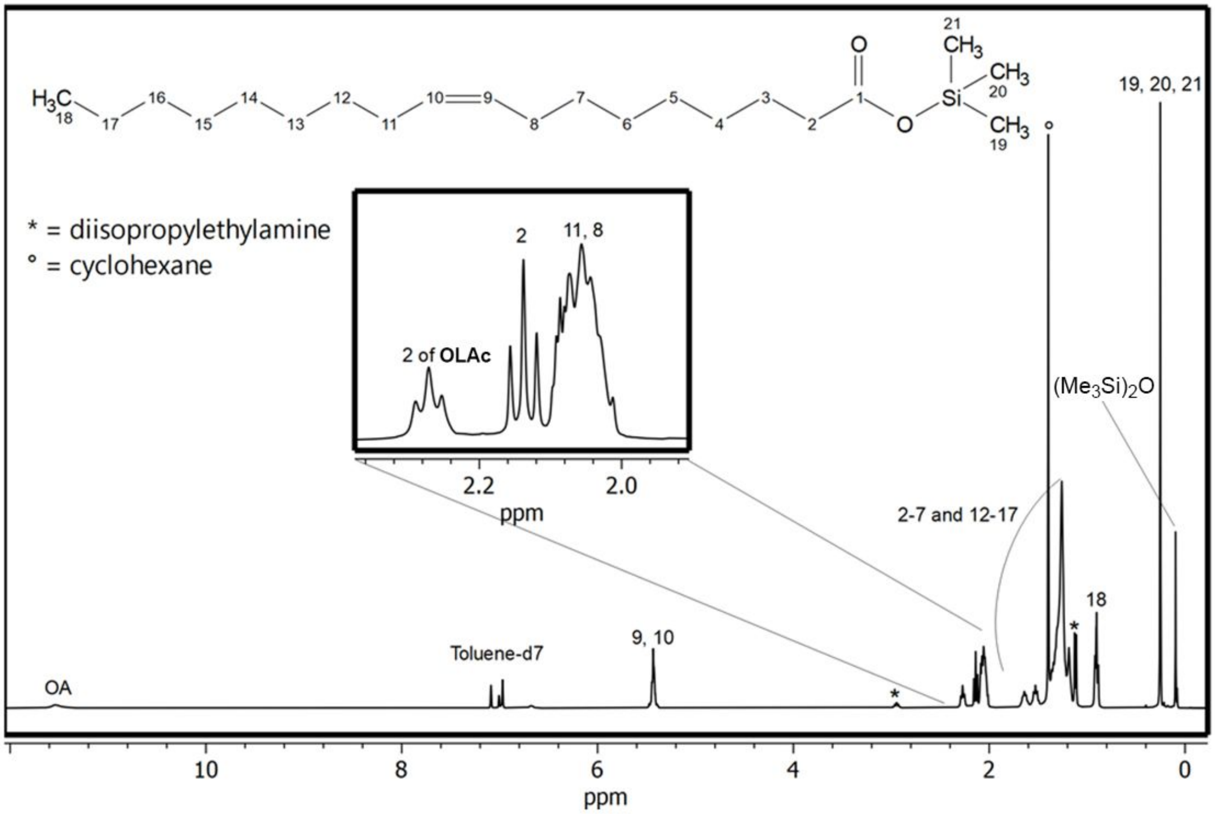

Figure S12. ^{1}H NMR spectrum of Me_3Si-Oleate in toluene-d, the structure and peaks assignment are embedded.

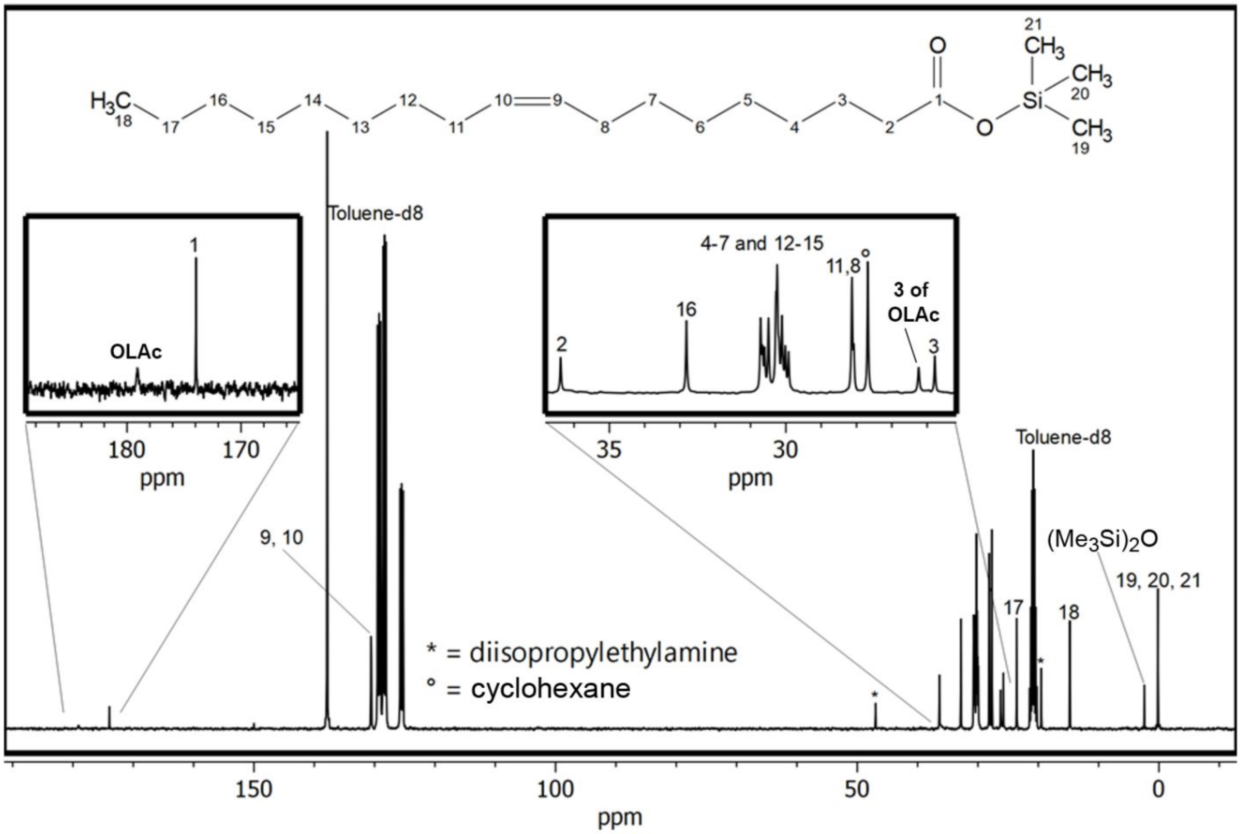

Figure S13. ^{13}C NMR spectrum of Me_3Si-Oleate in toluene-d, the structure and peaks assignment are embedded

The presence of a small amount of $(Me_3Si)_2O$ alongside Me_3Si-Oleate in the NMR spectra can be attributed to the hydrolysis of Me_3Si-based species or reactive Me_3Si intermediates by trace moisture present either in the reaction mixture or the solvent environment.

Procedure for the synthesis of N,N-dioctyl(trimethylsilyl)amine

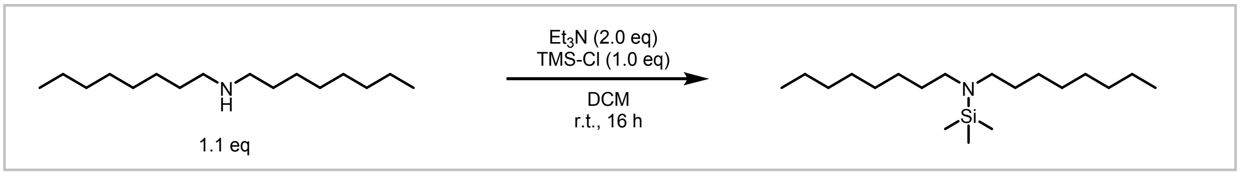

In a screwcap vial with septum, N,N-dioctylamine (0.3 mmol, 90 µL, 1.1 equivalents) was dissolved in dry Dichloromethane (DCM, 1.0 ml) under nitrogen atmosphere. Triethylamine (Et_3N, 0.6 mmol, 66 µL, 2.0 equivalents) was added followed by chlorotrimethylsilane (0.27 mmol, 34 µL, 1.0 equivalents). The reaction was left stirring overnight at room temperature. Cyclohexane was then added to the reaction mixture and the slurry was filtered. The volatiles were then removed, and the resulting orange oil was characterized without further purification. The product was stored in a -20°C freezer under nitrogen atmosphere.

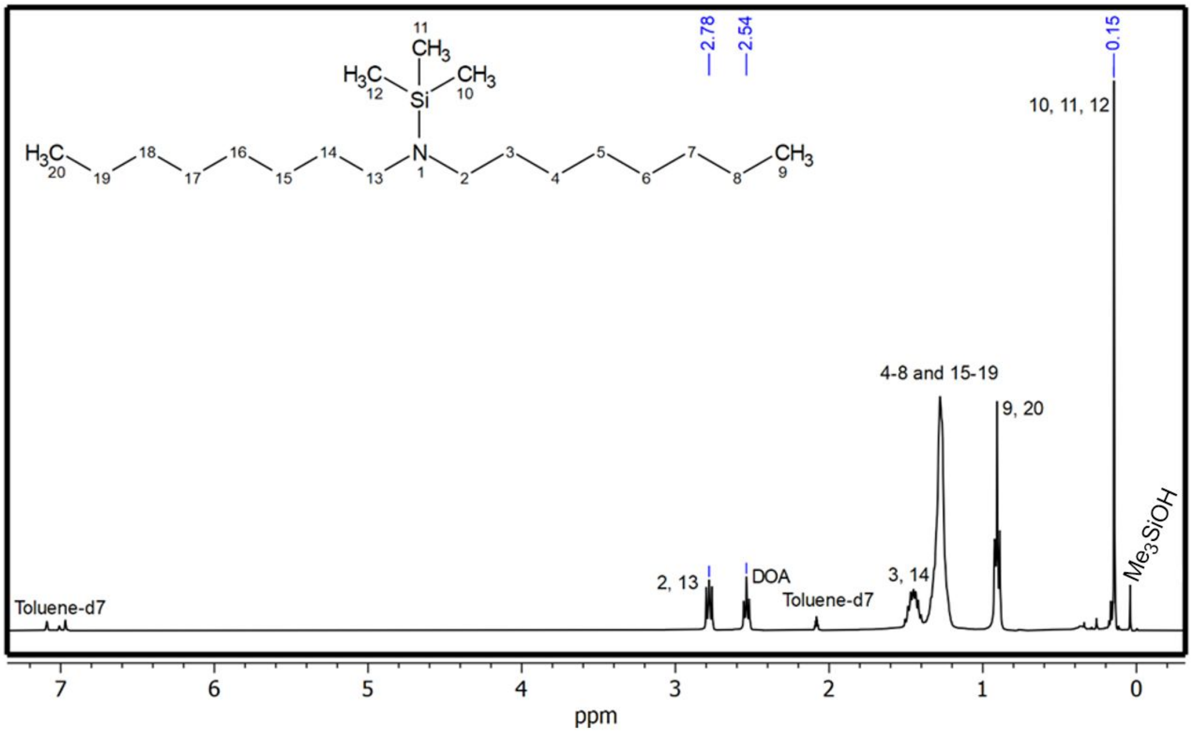

Figure S14. ^{1}H NMR spectrum of N,N-dioctyl(trimethylsilyl)amine in toluene-d, the structure and peaks assignment are embedded.

The NMR experiments were done in ambient conditions. Overtime, N,N-dioctyl(trimethylsilyl)amine was observed to readily decompose to form Me_3SiOH, as evidenced by the appearance of the characteristic peak of Me_3SiOH in **Figure S14**.

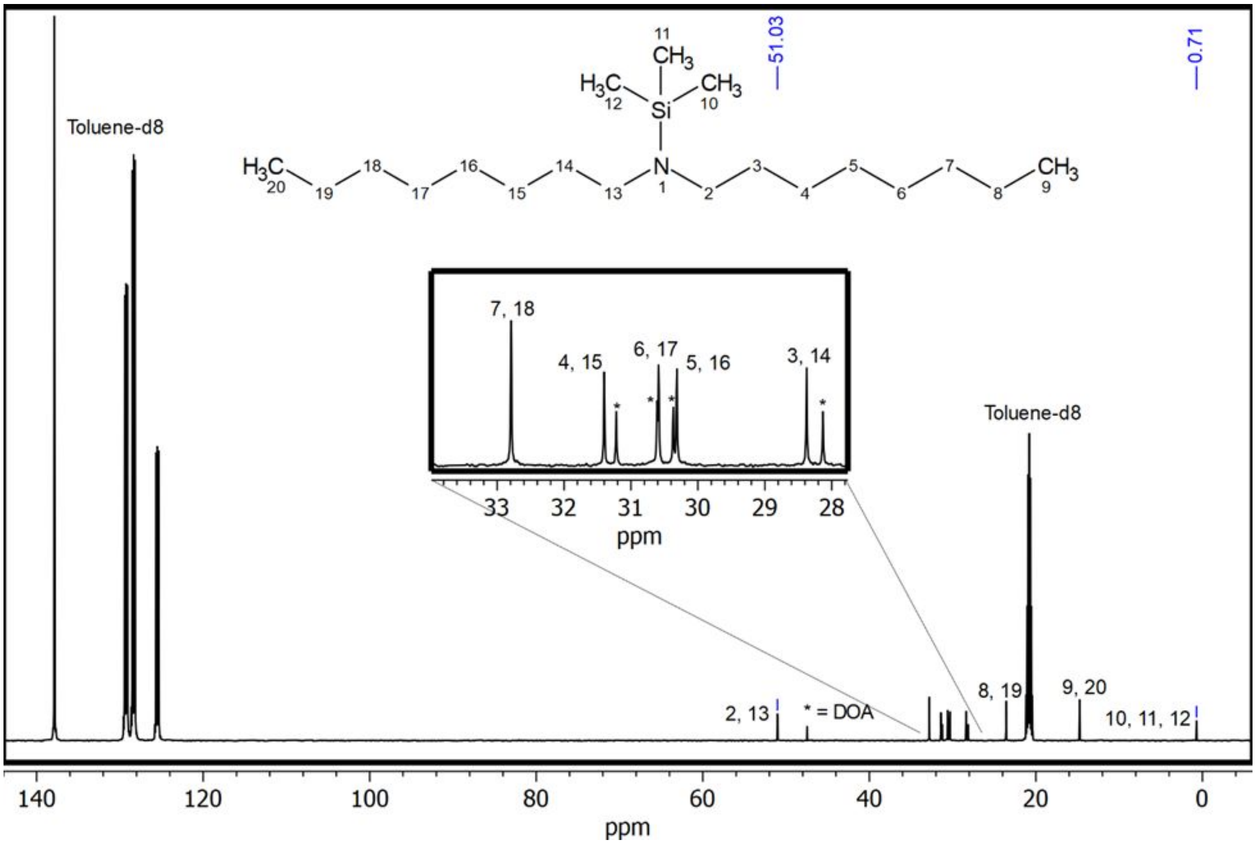

Figure S15. ^{13}C NMR spectrum of N,N-dioctyl(trimethylsilyl)amine in toluene-d, the structure and peaks assignment are embedded.

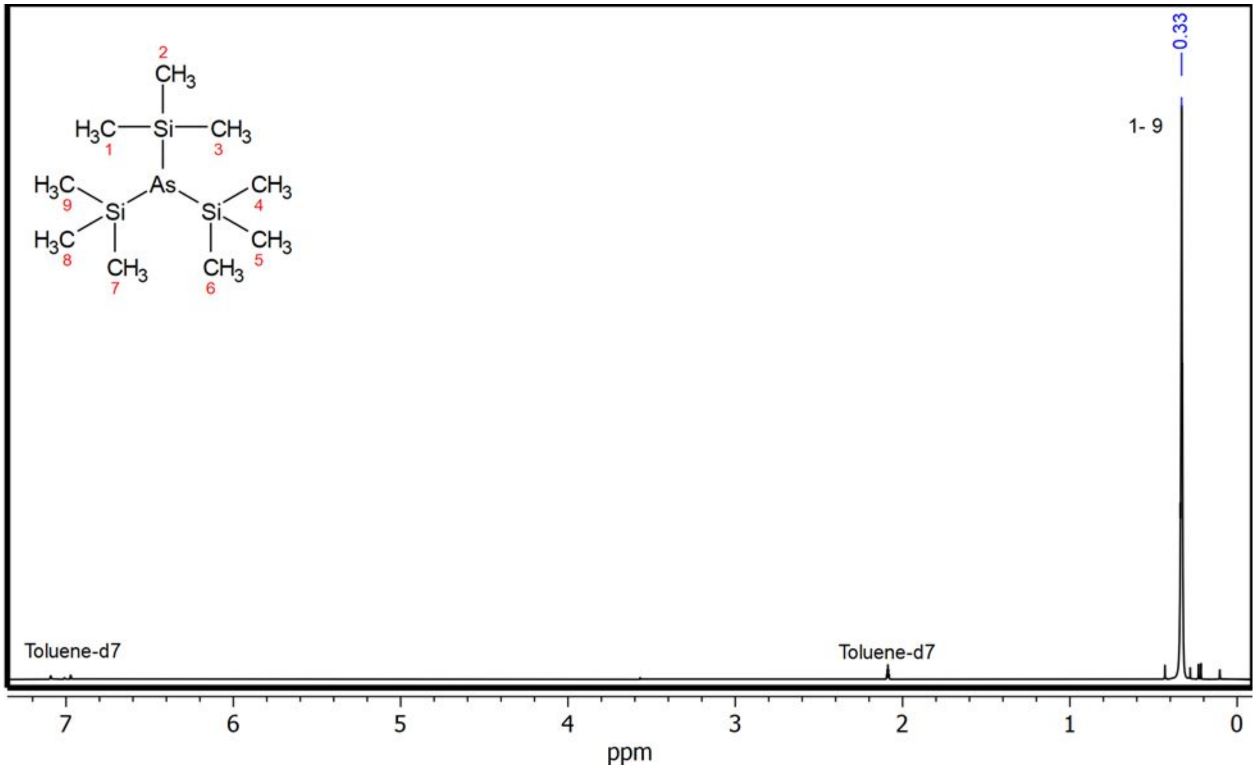

Figure S16. ^{1}H NMR spectrum of $(Me_3Si)_3As$ (Dock Chemicals, 99%) in toluene-d, the structure and peaks assignment are embedded.

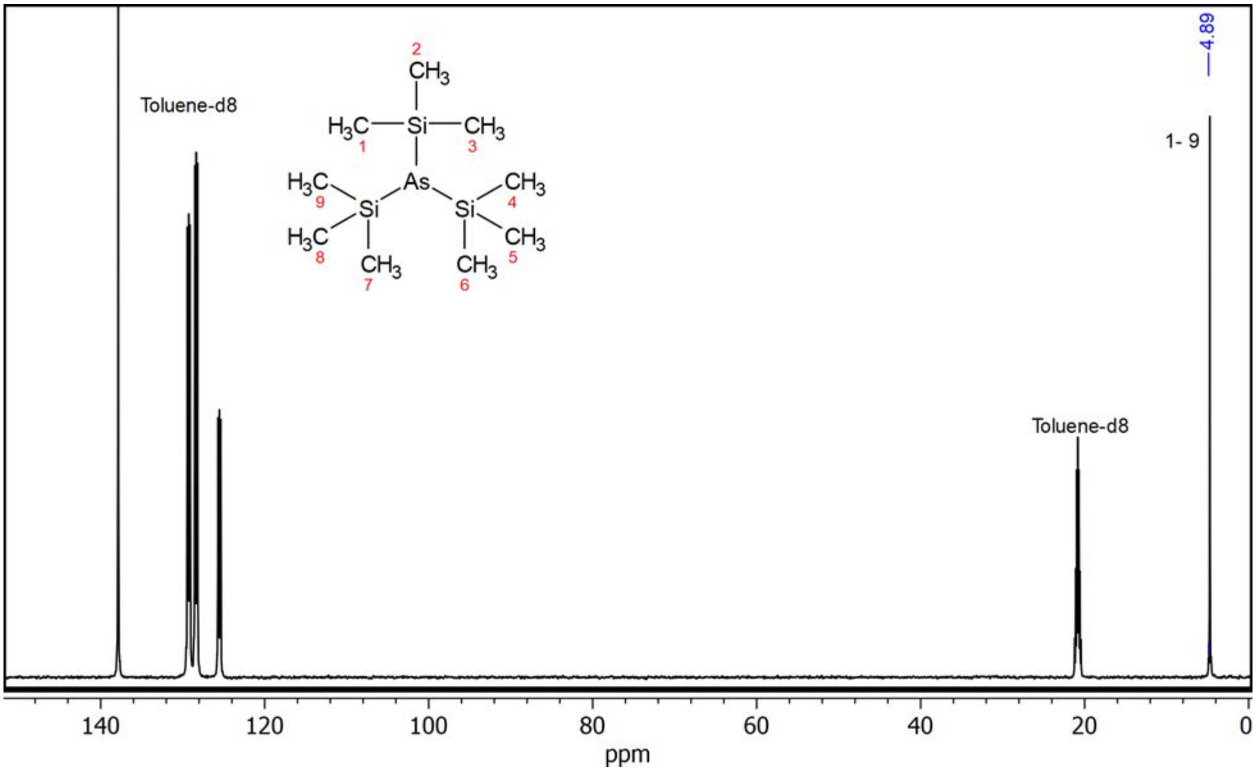

Figure S17. ^{13}C NMR spectrum of $(Me_3Si)_3As$ (Dock Chemicals, 99%) in toluene-d, the structure and peaks assignment are embedded.

$(Me_3Si)_3As$ + OLAc reaction: 2mL (6mmol) of OLAc was taken in a 25mL three-neck round bottom flask and degassed under vacuum at 120°C for 1 h. The temperature was then raised to 250°C under nitrogen, when 140µL (0.5mmol) of $(Me_3Si)_3As$ was injected into the reaction mixture. The reaction was allowed to proceed for 1h. After completion, 30µL of the reaction mixture was withdrawn and dispersed in toluene-d for subsequent NMR analysis.

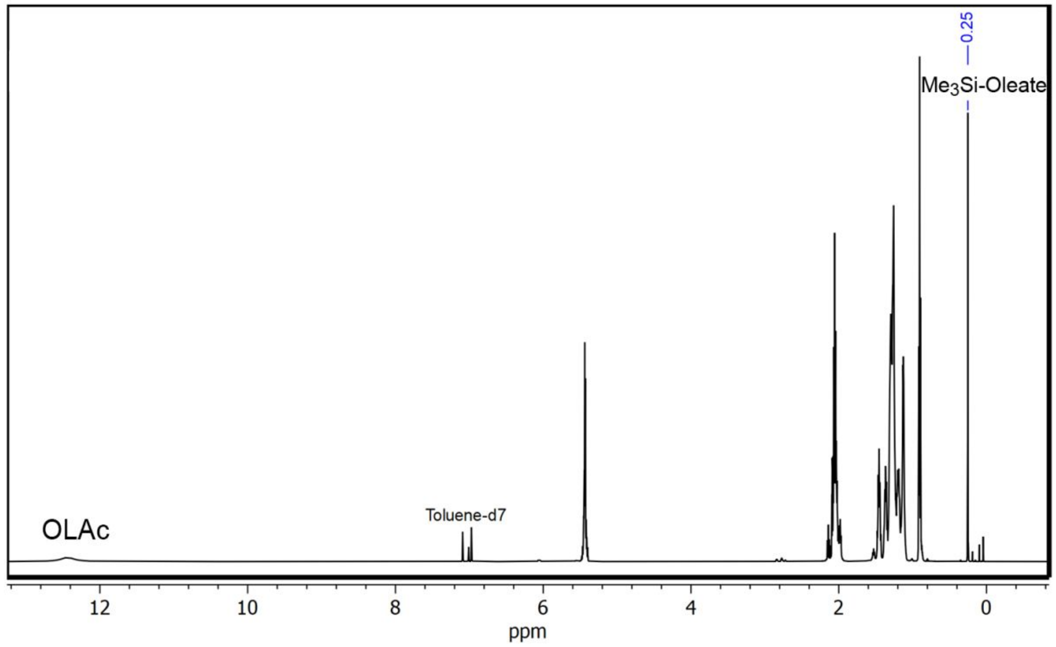

Figure S18. ^{1}H NMR spectrum of the reaction of $(Me_3Si)_3As$ with OLAc in toluene-d, diagnostic peaks are labeled.

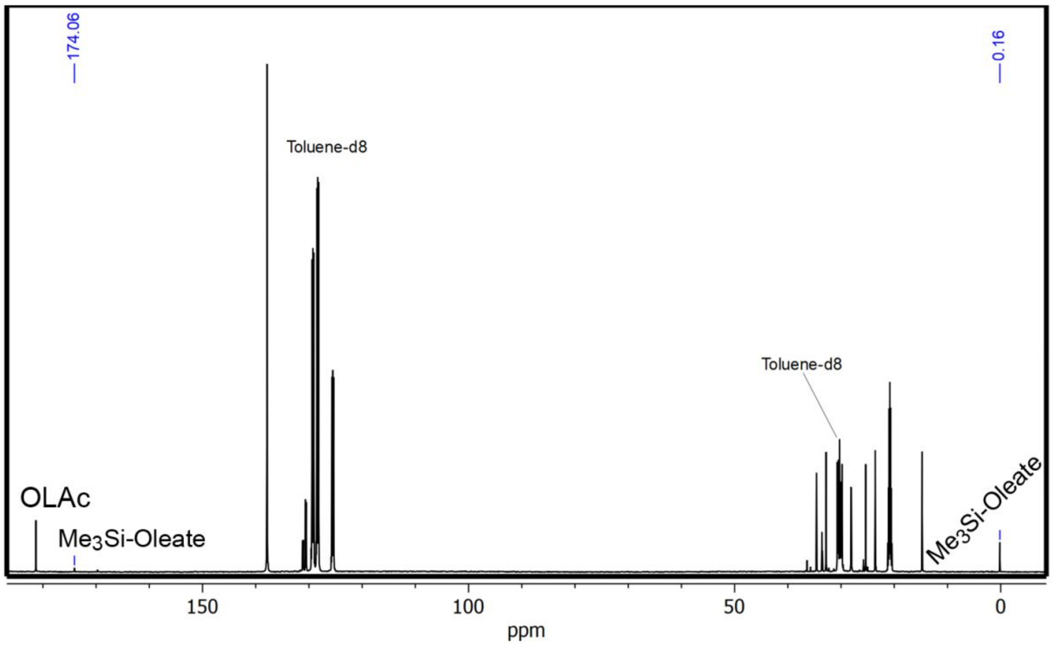

Figure S19. ^{13}CNMR spectrum of the reaction of $(Me_3Si)_3As$ with OLAc in toluene-d, diagnostic peaks are labeled.

Me_3SiOH + OLAc reaction: 2mL (6mmol) of OLAc was taken in a 25mL three-neck round bottom flask and degassed under vacuum at 120°C for 1 h. The temperature was then raised to 250°C under nitrogen, when 160μL of Me_3SiOH (1.5mmol) was slowly injected into the reaction mixture to avoid excessive boiling of Me_3SiOH. The reaction was allowed to proceed for 1h under nitrogen-flow. After completion, 30μL of the reaction mixture was withdrawn and dispersed in dry toluene-d for subsequent NMR analysis.

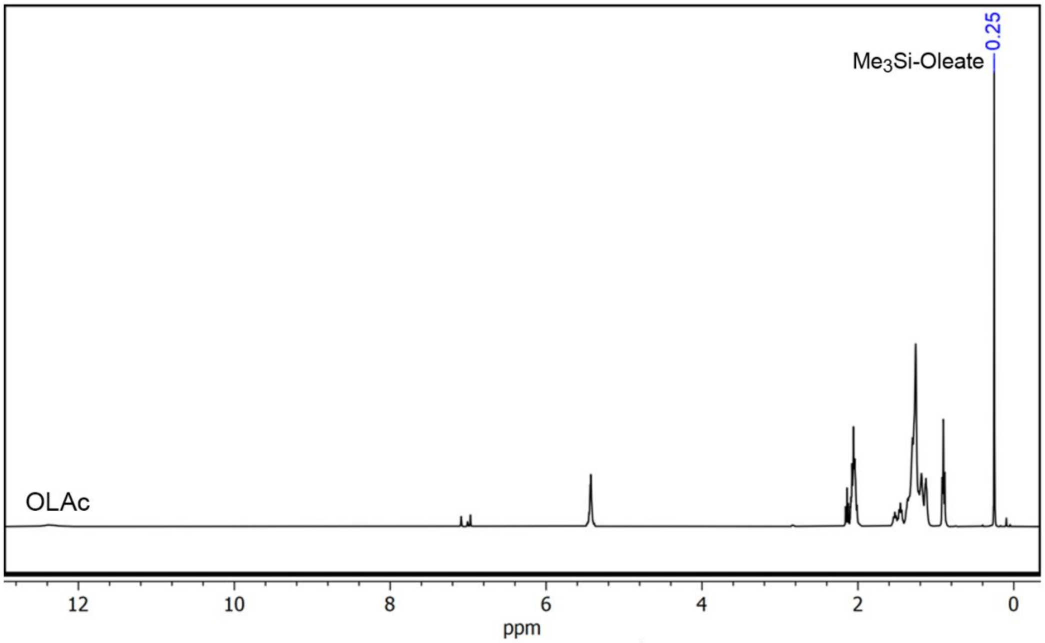

Figure S20. ^1H NMR spectrum of the reaction of Me_3SiOH with OLAc in toluene-d, diagnostic peaks are labeled.

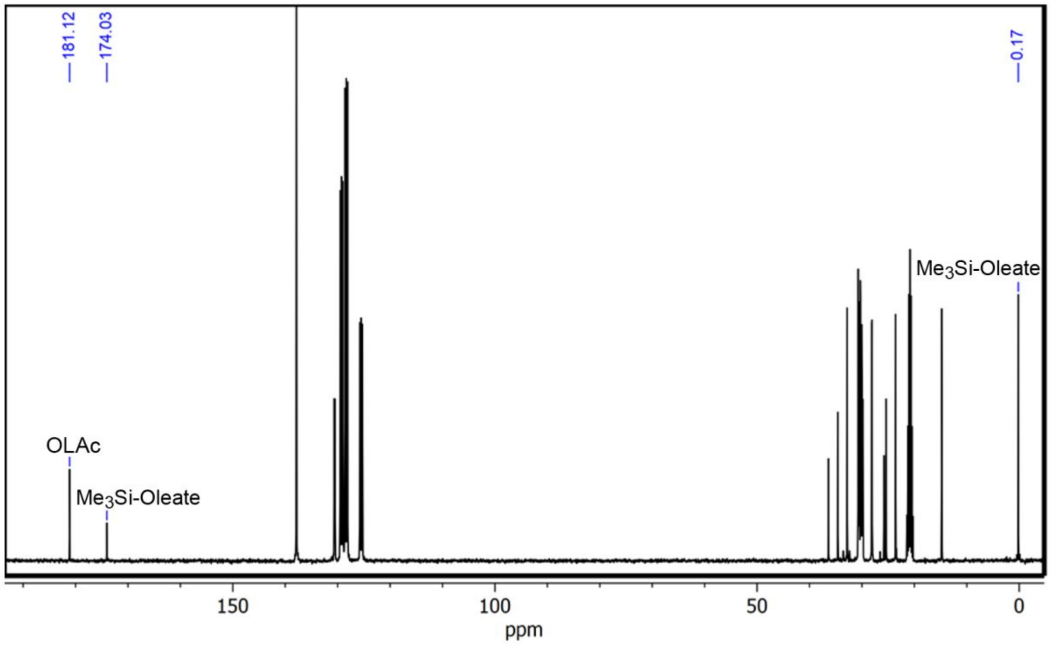

Figure S21. ^{13}C NMR spectrum of the reaction of Me_3SiOH with OLAc in toluene-d, diagnostic peaks are labeled

$(Me_3Si)_3As$ + DOA reaction: 900µL (3mmol) of DOA was taken in a 25mL three-neck round bottom flask and degassed under vacuum at 120°C for 1 h. The temperature was then raised to 250°C under nitrogen, when 140µL of $(Me_3Si)_3As$ (0.5mmol) was injected into the reaction mixture. The reaction was allowed to proceed for 1h under nitrogen. After completion, 30µL of the reaction mixture was withdrawn and dispersed in dry toluene-d for subsequent NMR analysis.

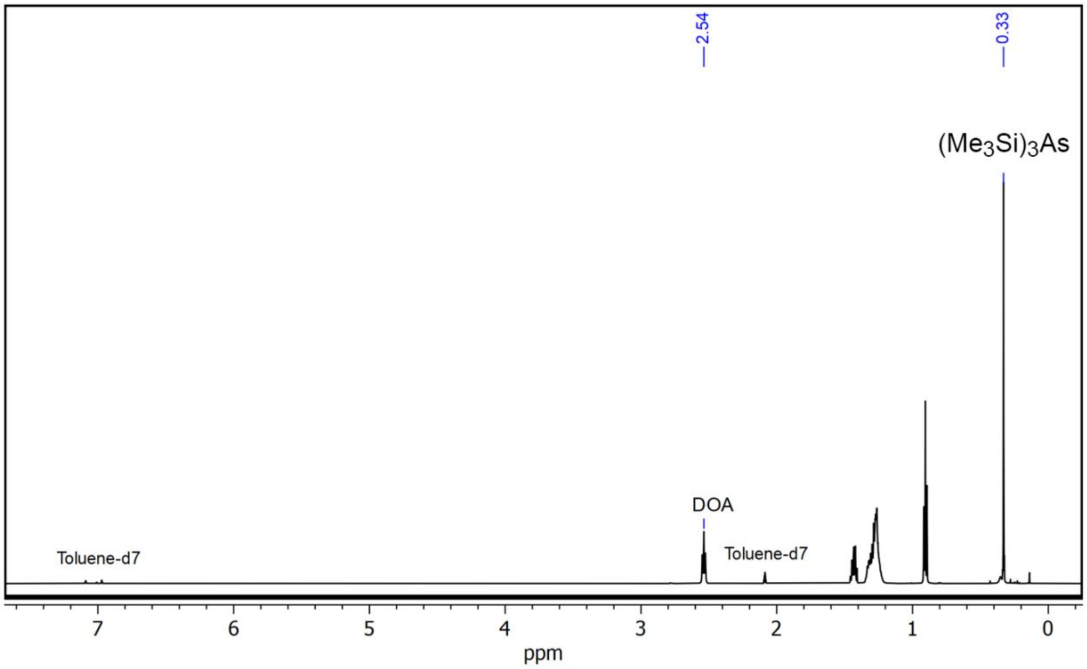

Figure S22. ^1H NMR spectrum of the reaction of $(Me_3Si)_3As$ with DOA in toluene-d, diagnostic peaks are labeled.

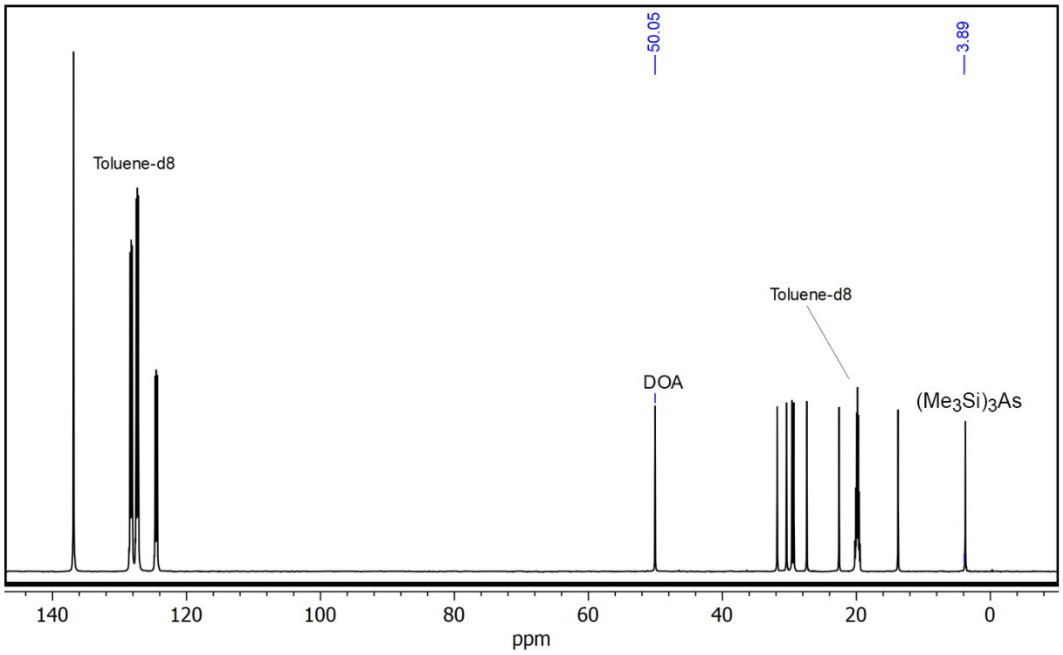

Figure S23. ^{13}C NMR spectrum of the reaction of $(Me_3Si)_3As$ with DOA, diagnostic peaks are labeled.

Me_3SiOH + DOA reaction: 900μL (3mmol) of DOA was taken in a three-neck round bottom flask and degassed under vacuum at 120°C for 1 h. The temperature was then raised to 250°C under nitrogen, when 160μL of Me_3SiOH (1.5mmol) was slowly injected into the reaction mixture to avoid excessive boiling of Me_3SiOH. The reaction was allowed to proceed for 1h under high nitrogen-flow. After completion, 30μL of the reaction mixture was withdrawn and dispersed in dry toluene-d for subsequent NMR analysis.

The Me_3SiOH +DOA reaction did not yield N,N-dioctyl(trimethylsilyl)amine, but mostly $(Me_3Si)_2O$ as a consequence of the reaction of Me_3SiOH with itself:

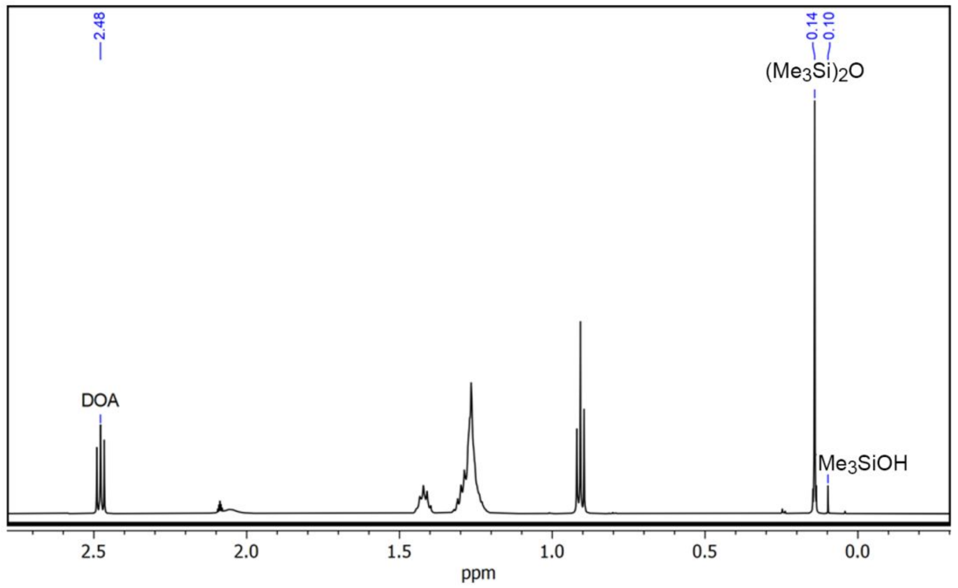

Figure S24. ^1H NMR spectrum of the reaction of Me_3SiOH with DOA in toluene-d, diagnostic peaks are labeled.

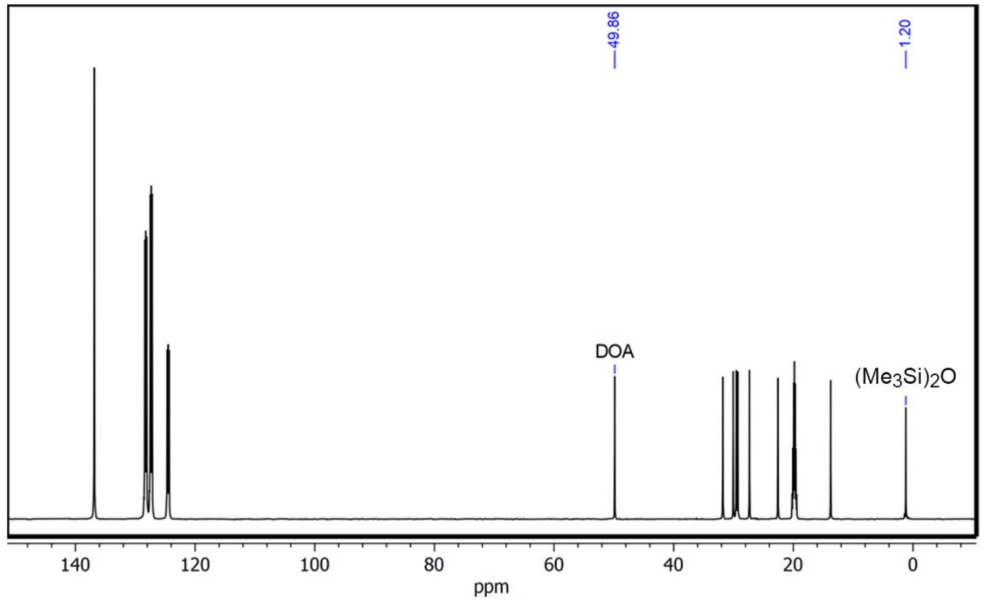

Figure S25. ^{13}C NMR spectrum of the reaction of Me_3SiOH with DOA in toluene-d, diagnostic peaks are labeled.

$(Me_3Si)_3As$ + OLAc + DOA reaction: 2mL (6mmol) of OLAc and 900µL (3mmol) of DOA was taken in a 25mL three-neck round bottom flask and degassed under vacuum at 120°C for 1 h. The temperature was then raised to 250°C under nitrogen, when 140µL of $(Me_3Si)_3As$ (0.5mmol) was injected into the reaction mixture. The reaction was allowed to proceed for 1h under nitrogen. After completion, 30µL of the reaction mixture was withdrawn and dispersed in dry toluene-d for subsequent NMR analysis.

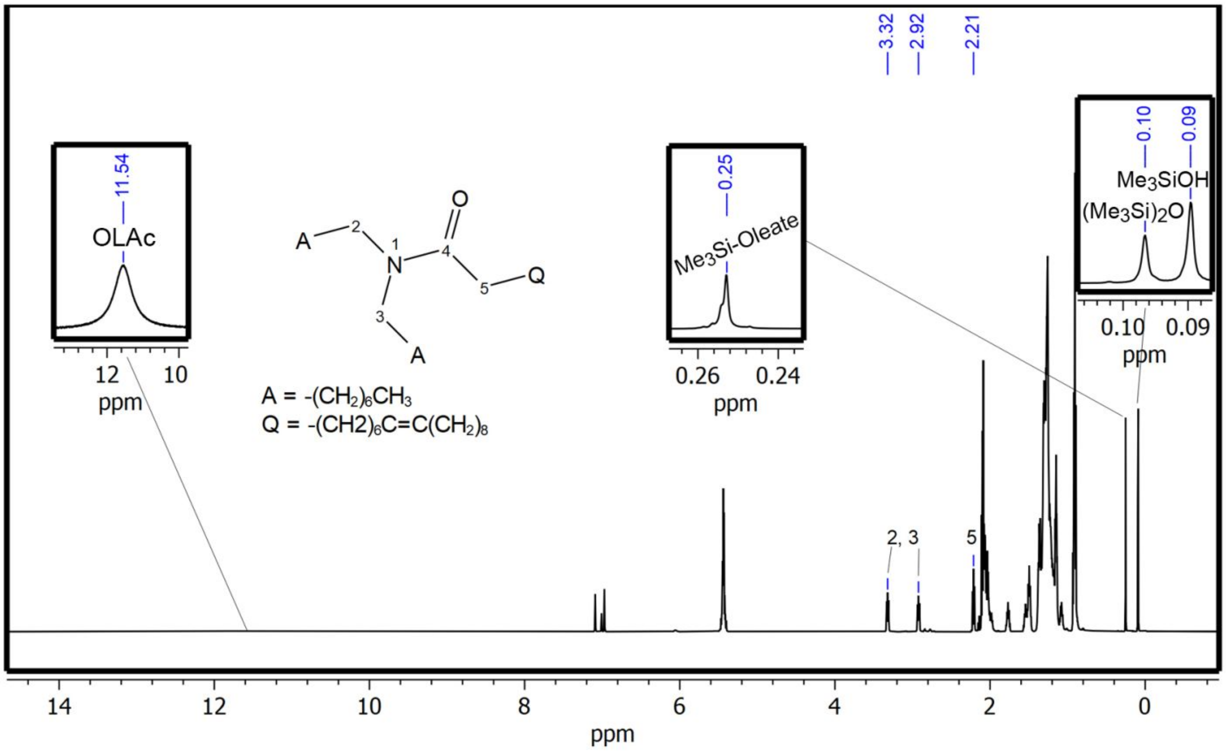

Figure S26. ^{1}H NMR spectrum of the reaction of $(Me_3Si)_3As$ with OLAc and DOA in toluene-d, diagnostic peaks are labeled. Peaks assignment was confirmed via spiking experiments (**Figure S29**)

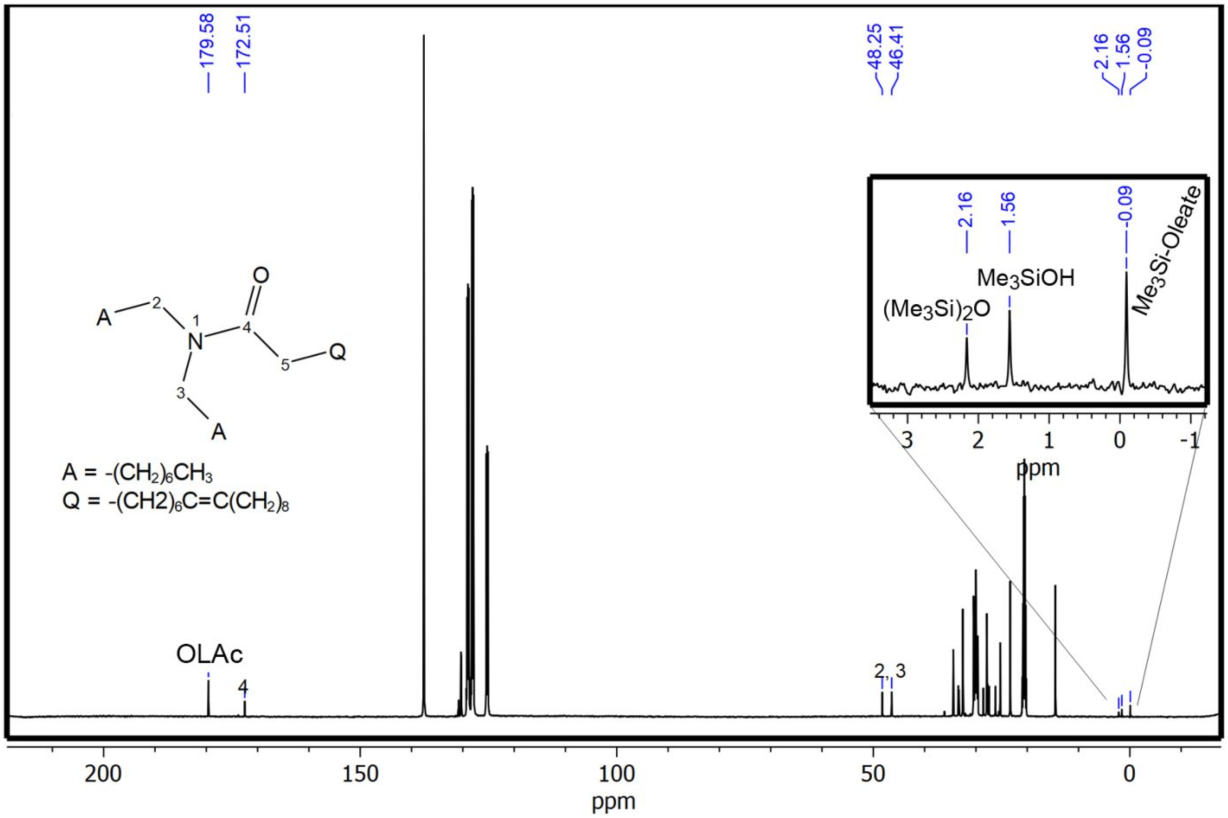

Figure S27. ^{13}C NMR spectrum of the reaction of $(Me_3Si)_3As$ with OLAc and DOA, diagnostic peaks are labeled.

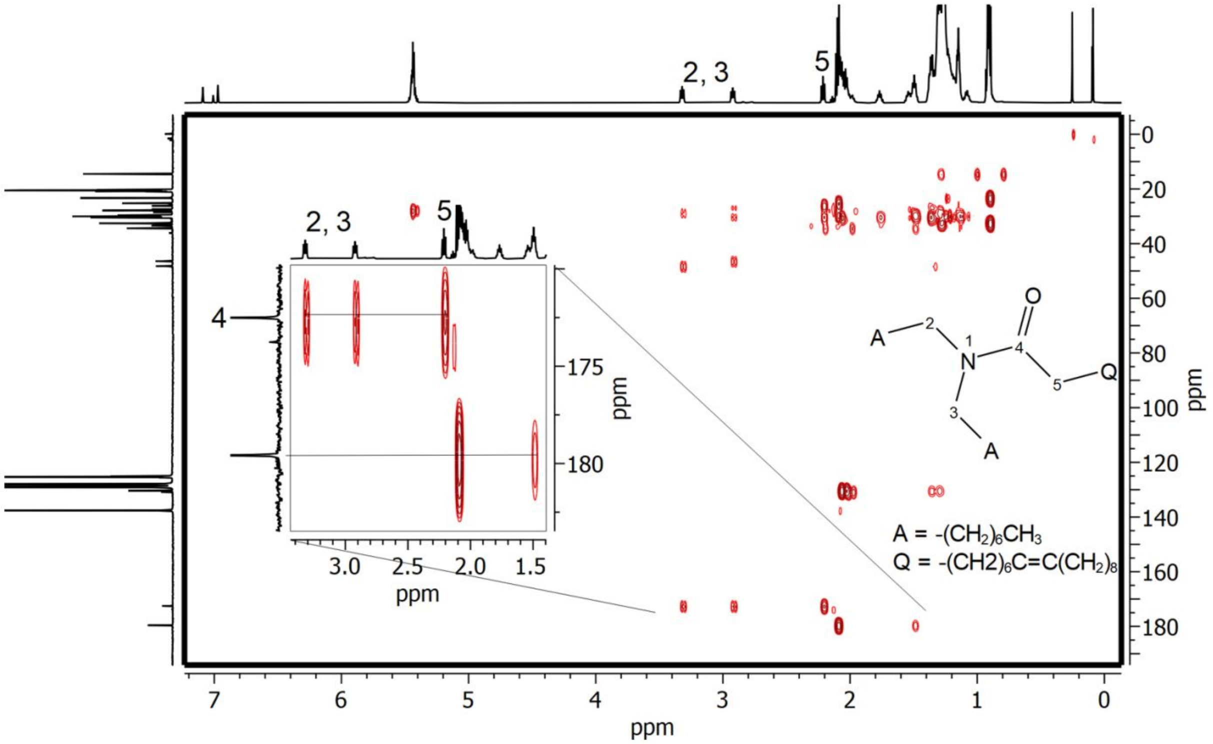

Figure S28. ^{1}H-^{13}C Heteronuclear Multiple Bond Correlation (HMBC) NMR spectrum of the reaction of $(Me_3Si)_3As$ with OLAc and DOA, as proof of the structure identity, the cross correlation HMBC peaks between both the CH_2 in position 2 and 3 and the one in position 5 with the carbon at 172.51 ppm, diagnostic of amide group, inset.

The ^{1}H-^{13}C HMBC experiment was recorded by using 16 FIDs, 4096 digit points, 128 increments, and $^1J_{CH}$ long range of 10 Hz, a spectral width of 15.15 ppm for ^{1}H and 220.00 ppm for ^{13}C, with a transmitter frequency offset at 7.00 and 100.00 ppm, respectively.

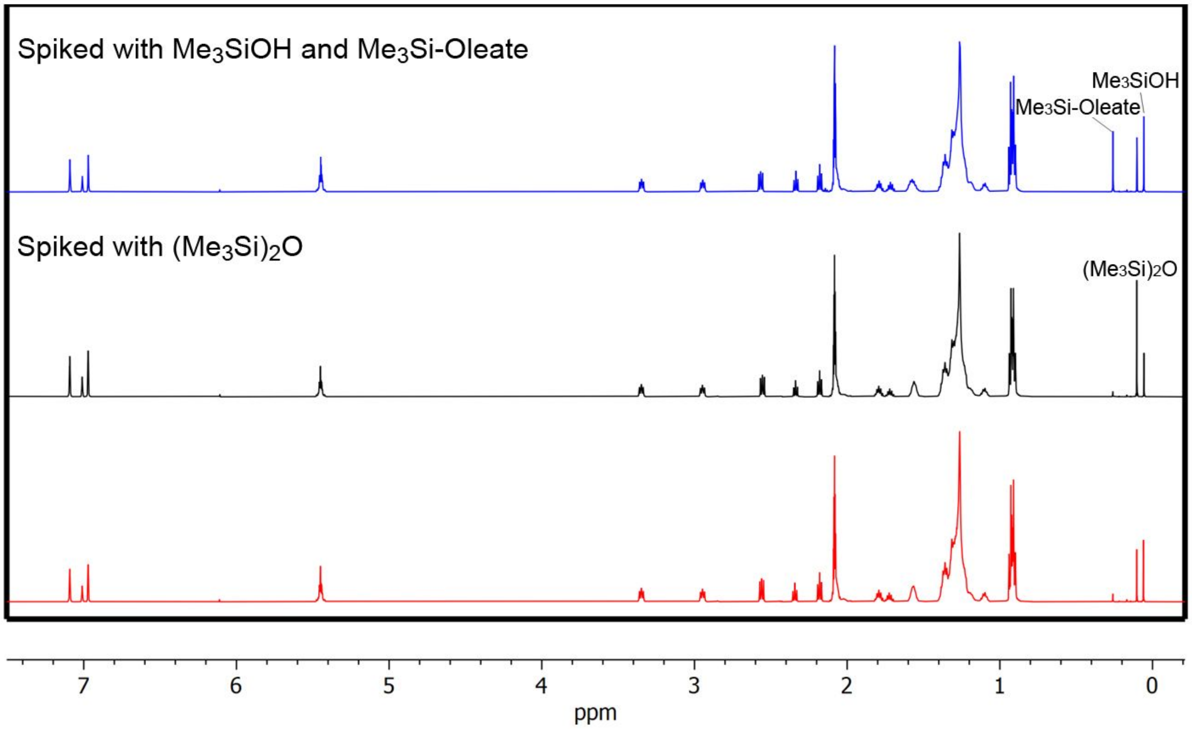

Figure S29. ^{1}H NMR spectrum of the reaction of $(Me_3Si)_3As$ +DOA+ OLAc (red spectrum), spiked with Me_3Si-Oleate, $(Me_3Si)_2O$ and Me_3SiOH authentic compound (black and blue spectra). Peaks increasing confirmed the attribution.

$(Me_3Si)_3As$ + TOA + OLAc reaction: 2mL (6mmol) of OLAc and 1.3mL (3mmol) of TOA was taken in a 25mL three-neck round bottom flask and degassed under vacuum at 120°C for 1 h. The temperature was then raised to 250°C under nitrogen, when 140µL of $(Me_3Si)_3As$ (0.5mmol) was injected into the reaction mixture. The reaction was allowed to proceed for 1h under nitrogen. After completion, 30µL of the reaction mixture was withdrawn and dispersed in dry toluene-d for subsequent NMR analysis.

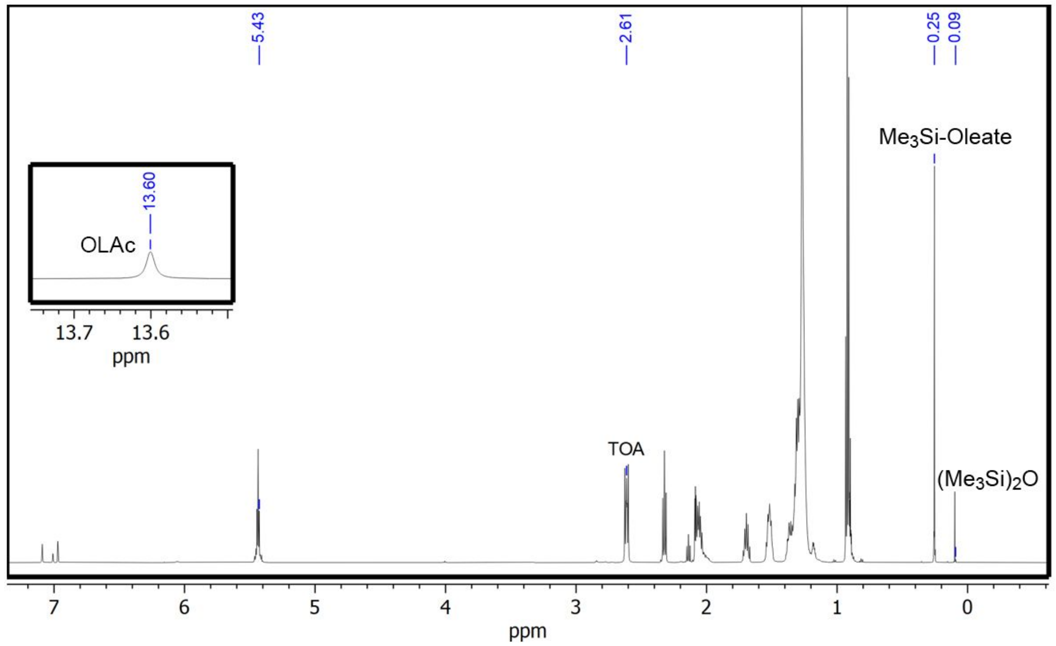

Figure S30. ^{1}H NMR spectrum of the reaction of $(Me_3Si)_3As$ with OLAc and TOA in toluene-d. Diagnostic peaks are labeled. Peaks assignment was confirmed via spiking experiments (**Figure S32**).

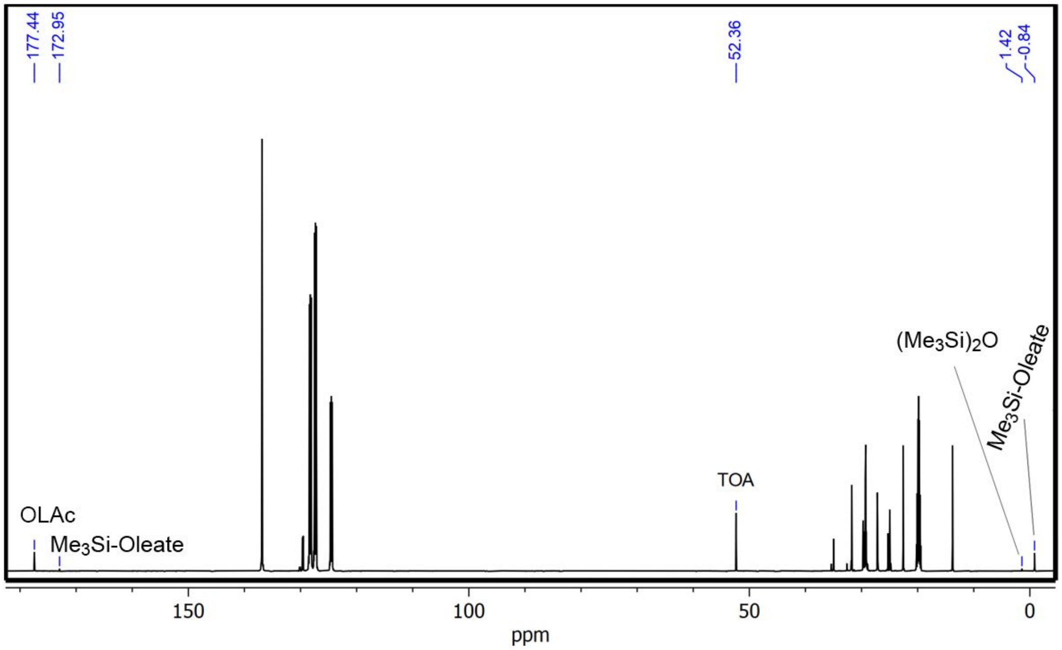

Figure S31. ^{13}CNMR spectrum of the reaction of $(Me_3Si)_3As$ with OLAc and TOA, diagnostic peaks are labeled

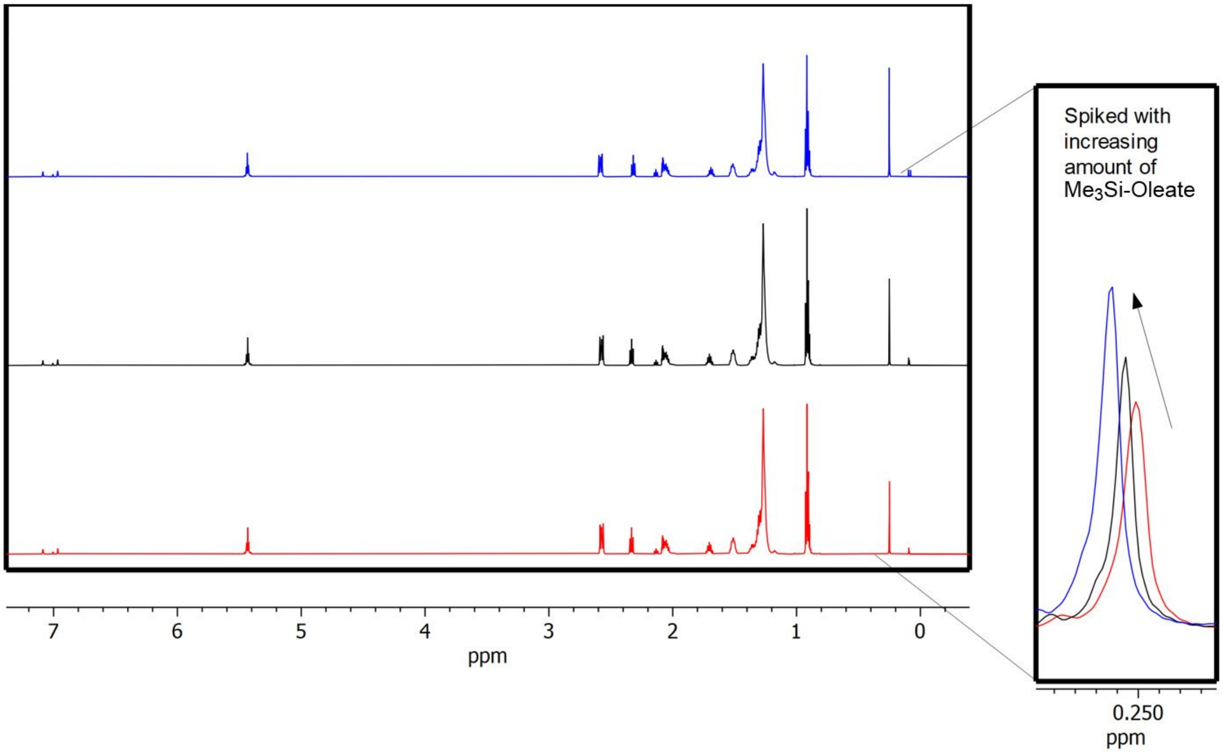

Figure S32. ^{1}H NMR spectrum of the reaction of $(Me_3Si)_3As$+TOA+OLAc (red spectrum), spiked with increasing amount of Me_3Si-Oleate authentic compound (black and blue spectra). The peak increasing confirm the peak assignment.

The presence of a small amount of $(Me_3Si)_2O$ alongside Me_3Si-Oleate in the products of the $(Me_3Si)_3As$ +TOA+OLAc reaction could be attributed to the hydrolysis of Me_3Si-based species or reactive Me_3Si intermediates by trace moisture present either in the reaction mixture or the solvent environment.

References

(1) Schneider, C. A.; Rasband, W. S.; Eliceiri, K. W. Nih Image to Imagej: 25 Years of Image Analysis. *Nature Methods* **2012**, *9*, 671-675, DOI: 10.1038/nmeth.2089.

(2) Song, J. H.; Choi, H.; Pham, H. T.; Jeong, S. Energy Level Tuned Indium Arsenide Colloidal Quantum Dot Films for Efficient Photovoltaics. *Nat. Commun.* **2018**, *9*, 4267, DOI: 10.1038/s41467-018-06399-4.

(3) Franke, D.; Harris, D. K.; Chen, O.; Bruns, O. T.; Carr, J. A.; Wilson, M. W. B.; Bawendi, M. G. Continuous Injection Synthesis of Indium Arsenide Quantum Dots Emissive in the Short-Wavelength Infrared. *Nat. Commun.* **2016**, *7*, 12749, DOI: 10.1038/ncomms12749.

(4) Tamang, S.; Lee, S.; Choi, H.; Jeong, S. Tuning Size and Size Distribution of Colloidal Inas Nanocrystals Via Continuous Supply of Prenucleation Clusters on Nanocrystal Seeds. *Chem. Mater.* **2016**, *28*, 8119-8122, DOI: 10.1021/acs.chemmater.6b03585.

(5) Henderson, Jeffrey D.; Pearson, L.; Nie, H.-Y.; Biesinger, Mark C. X-Ray Photoelectron Spectroscopy Analysis of Indium and Indium-Containing Compounds. *Surf. Interface Anal.* **2024**, *57*, 81-97, DOI: 10.1002/sia.7356.
